# Stratifying cellular metabolism during weight loss: an interplay of metabolism, metabolic flexibility and inflammation

**DOI:** 10.1038/s41598-020-58358-z

**Published:** 2020-02-03

**Authors:** Samar H. K. Tareen, Martina Kutmon, Theo M. de Kok, Edwin C. M. Mariman, Marleen A. van Baak, Chris T. Evelo, Michiel E. Adriaens, Ilja C. W. Arts

**Affiliations:** 1 Maastricht Centre for Systems Biology (MaCSBio), Maastricht University, Maastricht, The Netherlands; 2 Department of Bioinformatics – BiGCaT, NUTRIM School for Nutrition and Translational Research in Metabolism, Maastricht University, Maastricht, The Netherlands; 3 Department of Toxicogenomics, GROW School of Oncology and Developmental Biology, Maastricht University, Maastricht, The Netherlands; 4 Department of Human Biology, NUTRIM School for Nutrition and Translational Research in Metabolism, Maastricht University, Maastricht, The Netherlands; 5 Department of Epidemiology, CARIM School for Cardiovascular Diseases, Maastricht University, Maastricht, The Netherlands

**Keywords:** Computational biology and bioinformatics, Systems biology

## Abstract

Obesity is a global epidemic, contributing significantly to chronic non-communicable diseases, such as type 2 diabetes mellitus, cardiovascular diseases and metabolic syndrome. Metabolic flexibility, the ability of organisms to switch between metabolic substrates, is found to be impaired in obesity, possibly contributing to the development of chronic illnesses. Several studies have shown the improvement of metabolic flexibility after weight loss. In this study, we have mapped the cellular metabolism of the adipose tissue from a weight loss study to stratify the cellular metabolic processes and metabolic flexibility during weight loss. We have found that for a majority of the individuals, cellular metabolism was downregulated during weight loss, with gene expression of all major cellular metabolic processes (such as glycolysis, fatty acid *β*-oxidation etc.) being lowered during weight loss and weight maintenance. Parallel to this, the gene expression of immune system related processes involving interferons and interleukins increased. Previously, studies have indicated both negative and positive effects of post-weight loss inflammation in the adipose tissue with regards to weight loss or obesity and its co-morbidities; however, mechanistic links need to be constructed in order to determine the effects further. Our study contributes towards this goal by mapping the changes in gene expression across the weight loss study and indicates possible cross-talk between cellular metabolism and inflammation.

## Introduction

Obesity has, in recent years, been classified as a global epidemic. WHO estimates have shown a steady increase in obesity with trends expected to continue^1^. This makes obesity a public health concern as it has been linked to cardiovascular diseases, type 2 diabetes and metabolic syndrome^2–4^. Therefore, weight loss and exercise is encouraged to counter obesity and, by extension, to prevent the progression of its co-morbidities^5,6^. A focus in recent research has been to find the mechanistic links between obesity and associated co-morbidities by studying the underlying molecular processes^7,8^. In one of our previous studies^9^, we explored the expression of genes changing in the subcutaneous adipose tissue over time in response to caloric restriction. We observed that one of the clusters of co-expressed genes, which was being differentially expressed or differentially regulated over caloric restriction, was involved with a number of processes associated with the tricarboxylic acid (TCA) cycle and cellular metabolism. Interestingly, these processes are also part of what collectively constitutes the system of metabolic flexibility – the ability of organisms to switch metabolic substrates depending on nutrient conditions and needs^10^. In our study, this amounted to switching between glucose metabolism and fatty acid metabolism as these are the chief metabolic substrates for the majority of the tissue and cell types in the human body^11^.

Studies have found an association between impaired metabolic flexibility (also known as metabolic inflexibility) and type 2 diabetes mellitus, cardiovascular diseases and metabolic syndrome^12–15^. Other studies have narrowed down the effects of metabolic inflexibility in the adipose tissue on the impairment of adipokine signalling as well as the clearance of circulating non-esterified fatty acids (as reviewed in^11,16^). Corpeleijn *et al*. showed an improvement in metabolic flexibility after weight loss, by an increase in fatty acid uptake and oxidation in skeletal muscles, indicating a positive association between the two^17,18^. Yet, several studies also show that formerly obese individuals exhibit metabolic inflexibility post weight loss in response to high-fat diets^19^. There is also evidence that individuals may be genetically predisposed to be more or less metabolically flexible^19^ although the extent of this predisposition is not currently well established.

In this article, we utilise a metabolic flexibility gene set based on cellular metabolism to cluster transcriptomics and proteomics expression data from a weight loss study. The purpose of this exercise is to identify and analyse expression profiles of individuals clustered by cellular metabolism centring on metabolic flexibility. In our previous studies^9,20,21^, we have shown how the cellular regulation of the tricarboxylic acid cycle, as well as the switching of substrates between glucose and fatty acids have a central role to play in obesity and chronic ailments such as type 2 diabetes mellitus and metabolic syndrome. Capitalising on this work, we curated a list of 291 genes involved in, or associated with the regulation of, cellular metabolism and substrate switching. The data was then clustered on samples of the individuals to identify pathways and processes associated with the change in metabolic profiles.

## Materials and Methods

### Dataset

We used expression data from the ‘Yoyo study’^22^ (Clinical Trial ID: NCT01559415, www.clinicaltrials.gov), a human weight loss study exploring the yo-yo effect seen as subsequent weight regain. The study covers two diets: a low calorie diet (LCD) of 1,250 kcal/day and a very low calorie diet (VLCD) of 500 kcal/day. The study design has four time points for data collection: the first being before dietary intervention (time point 1), followed by one at the end of weight loss diet (12 weeks for LCD, 5 weeks for VLCD) (time point 2), a third after a 4-week weight maintenance period (time point 3), and follow-up 9 months later (time point 4). All the participants of the study were Caucasian with a BMI between 28 kg/m^2^ and 35 kg/m^2^, aged between 32 and 67 years old (median age of 51 years). Additionally, the amount of weight lost by the individuals in both diets was almost the same. For further details, we refer to the original study publication^22^.

#### Transcriptomics

The Yoyo study has microarray transcriptomics measurements for 46 individuals for the first three data collection points of the study, with samples from the subcutaneous adipose tissue of the individuals. The transcriptomics data is available on Gene Expression Omnibus under ID: GSE77962. The array platform is Affymetrix Human Gene ST 1.1 arrays.

In our previous analysis^9^ we have analysed and normalised the transcriptomics data where we removed outliers, reducing the number of individuals to 44. We have also performed background noise correction on the measurements by removing all genes with median expression equal to or less than the expression of Y-chromosome genes in female individuals, giving us measurements for a total of 18,113 genes/transcripts.

#### Proteomics

The proteomics data from the Yoyo study are from the adipose tissue samples at time points 1 through 3 using liquid chromatography-mass spectrometry (LC-MS/MS)^23^. Both the samples and controls were labelled with TMT isobaric mass tagging labelling reagent (10plex, Thermo Scientific) and measured in the Dionex ultimate 3000 nanoflow HPLC instrument. The mass spectrometry data was then queried against UniProt using Sequest HT Proteome Discoverer 2.1 (Thermo Scientific)^23^. In this study, we use the corrected proteomics data (corrected for between and within runs) generated in the original study, and refer the readers to it for details regarding the parameters of the data generation. The proteomics data is freely available from the authors upon request.

#### Phenotypic measurements

The Yoyo study includes phenotypic and clinical measurements of the individuals. The anthropometric measurements^22^ include sex, age, height, weight, body mass index (BMI), fat mass, fat-free mass, hip circumference, waist size, systolic and diastolic blood pressure, and physical activity score (calculated via the Baecke questionnaire for habitual physical activity^24^). The anthropometric measurements, apart from age and height, were measured at each data collection point in the original study.

Likewise, fasting forearm venous plasma^25^ concentrations of glucose, insulin, free fatty acids, triglycerides and total cholesterol levels, and HOMA-IR index were measured for each data collection point. The adipose tissue arteriovenous flux measurements^26^, however, were measured only in a subset of individuals (13 of the 38). These measurements were taken at time points 1 and 3, and included flux measurements for glucose, free fatty acids, total glycerol, free glycerol, triacylglycerol and lactate, as well as measurements of total blood flow, plasma blood flow and insulin. An additional measurement available from the Yoyo study is the s-value, which is a score for weight regain and/or maintenance defined as the ratio of weight loss maintained at follow up to the weight lost immediately following intervention. Mathematically, this is represented as s-value = (W_*T*4_ − W_*T*3_)/(W_*T*2_ − W_*T*1_) where W_*Tn*_ is the weight of the individual at time point *n*. The phenotypic measurements described here are also freely available from the authors upon request.

### Metabolic flexibility gene list

In a previous study^20^, we explored the cellular processes involved in cellular metabolic flexibility and presented them as a combined network of rate limiting steps involved in these processes. In the current study, we utilised the network and processes from this review to curate a list of genes/proteins involved in the regulation of cellular metabolic flexibility. This list is provided as Supplementary file 1 and consists of 291 genes/proteins.

### Affinity network fusion

For the clustering of the transcriptomics and proteomics expression data across time points, we utilise affinity network fusion (ANF)^27^. ANF itself is built on top of the similarity network fusion algorithm^28^. These methods utilise multi-omics data to construct clusters of individuals that are more similar to each other than to others across their data spectrum. ANF is an optimisation over the original algorithm and incorporates weights for the various sources of the multi-omics data when constructing the affinity network to use with spectral clustering.

In our study, we utilised ANF to generate clusters of samples based on the transcriptomics and proteomics expression data filtered on the genes/proteins in the metabolic flexibility gene list. 38 individuals were used in the clustering totalling 106 samples across the three time points; 8 individuals were missing one measurement each at any of the three time points. The expression data used by the ANF algorithm consisted of the transcriptomics with 240 genes measured out of the 291 initially selected, and the proteomics with only 27 proteins. As the usable proteomics data were much smaller in amount and much larger in dynamic range compared to the transcriptomics, we opted to weight the two expression data types as the percentage coverage of the metabolic flexibility gene list, i.e., 240/291 for transcriptomics and 27/291 for proteomics.

The ANF algorithm also uses the k-nearest neighbour parameter to construct robust affinity networks. In our study, we tested k = {3, 4, 5, 6, 7, 8, 9, 10} as the possible nearest neighbour values. In all cases, the algorithm produced two clusters, with only one sample changing cluster membership from k = 8 onwards. The cluster membership list is provided as Supplementary file 2. We chose the clustering at k = 3 for further analysis due to the robustness of the clustering given that the cluster membership did not change for several increasing k-values after 3.

### Statistical analysis of phenotypic measurements

The means of the various phenotypic measurements of the respective samples of each cluster were compared using t-tests in R^29^, whereas a chi-square test was used for the comparison of sex. The obtained p-values were corrected for multiple testing using the Benjamini & Hochberg approach, that controls the false discovery rate (FDR)^30^.

### Differential expression analysis

For the differential expression analysis between samples of the clusters at different time points, we used the whole transcriptomics data set analysed via Limma v3.40.2^31^ package in R v3.5.1^29^. The criteria for defining differentially expressed genes was |Fold change| ≥ 1.2 and p-value < 0.05^32^. For differential comparisons between samples from the same set of individuals, the individual was used as a blocking factor in the linear model fit. For comparisons between sets with a mixture of unique and repeated individuals, the correlation between samples from the same individuals was modelled using the duplicate correlation function in limma.

### Gene ontology enrichment

Gene ontology enrichment was performed in Cytoscape v3.7.1^33^ using the ClueGO app for Cytoscape v2.5.4^34^. Enrichment was performed for biological process ontologies (updated: 10-04-2019) with all evidences used for input except for ‘inferred from electronic annotation’ (All_without_IEA checkbox). Furthermore, Gene Ontology term fusion was allowed and pathway selection was restricted to pathways with p-value ≤ 0.05. All other parameters in ClueGO remained the same.

### Pathway overrepresentation analysis

For pathway overrepresentation analysis, we used PathVisio v3.3.0^35^. PathVisio is a software that uses expression data to map and visualise various pathways from the WikiPathways database^36^ while also using z-score to analyse which pathways are overrepresented in the database based on the mapped expression data. This allows us to focus on pathways in relevant biological context to infer the meaning behind the expression data. In this study, the pathway selection criteria used was a z-score ≥ 1.96 (critical score for 95% confidence interval), a p-value < 0.05 and at least three significantly changed genes (significance criteria the same as in differential expression analysis). For a tutorial on PathVisio, we redirect the reader to^37^.

## Results

### Stratification of the metabolic profiles

**Figure 1:**
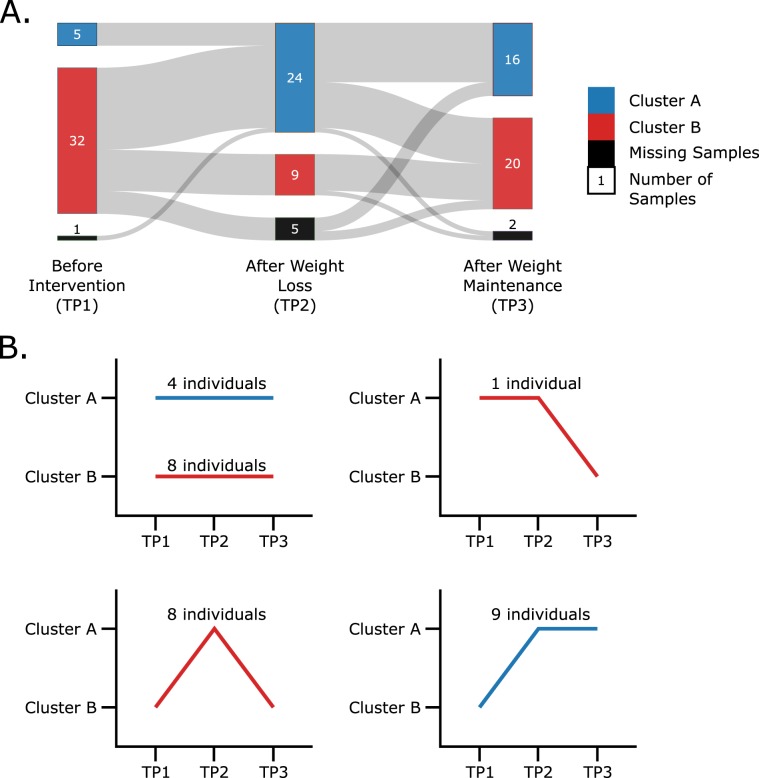
(**A**) Sankey diagram showing the proportion of individuals changing or not changing clusters across the time points of the dietary intervention. Eight individuals had missing samples at different time points and thus, were not clustered at those time points. These samples are shown as black blocks at the respective time points. (**B**) The various cluster membership patterns of the thirty individuals observed across the three time points, as well as the respective number of individuals following said pattern.

Using the affinity network fusion (ANF) algorithm for clustering we generated a two-cluster system with samples across individuals and time points clustered with each other. The primary purpose of this clustering was to cluster the transcriptomics and proteomics expression patterns of the 291 metabolic flexibility related genes and proteins. The clustering thus generated two expression profiles, with individuals changing cluster membership over the duration of the study (visualised in Fig. 1A). Figure 1B shows the respective individuals in each cluster membership pattern found. In this subfigure, we can see that at the beginning of the caloric restriction (TP1) the majority of individuals are in Cluster B compared to Cluster A. Immediately after weight loss, at time point 2 (TP2), the cluster membership for Cluster A surges to become larger than Cluster B, with both clusters settling close to even after weight maintenance (TP3). This indicates that the metabolic profile of a large number of individuals changed during weight loss with some of them maintaining that change whereas others reverted to their original profile.

We next compared the phenotypic/clinical measurements between the clusters with 101 samples instead of 106 due to missing/incomplete data for two individuals. We identified significant differences in sex, height, BMI, fat mass, fat free mass, waist size and hip circumference. From the forearm venous plasma measurements, total cholesterol, insulin, free fatty acids, triglycerides levels as HOMA-IR index were found to be significantly different between the clusters. However, none of the adipose tissue arteriovenous flux measurements were found to be significant post multiple testing correction. Lastly, the s-value, defined as a value for weight regain and/or maintenance, was also not found to be significantly different between the two clusters. These results are collectively provided as Supplementary file 3.

**Figure 2:**
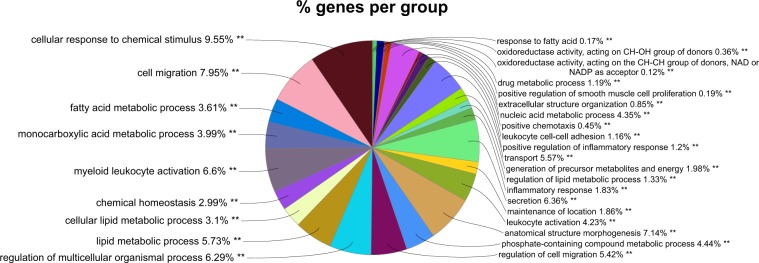
Pie chart showing the proportions of genes enriched for each of the gene ontology term for the differentially expressed genes between Cluster A and B. The differentially expressed genes were significant at p-value < 0.05 and an absolute fold change of at least 1.2.

Differential expression analysis of the 18,113 gene transcriptomics data between the samples of the two clusters showed that only 1,343 genes were differentially expressed between the two clusters (|Fold change| ≥ 1.2 and p-value < 0.05). 150 of these differentially expressed genes had an absolute fold change greater than 1.5, and 28 of those had it greater than 2. The number of differentially expressed genes have been tabulated in Table 1. A gene ontology enrichment analysis^34^ on the 1,343 genes revealed that apart from the expected metabolic processes (fatty acid and other lipid metabolism, oxidoreductase activity, chemical homoeostasis etc.) there were processes related to tissue morphology and inflammatory response also being enriched. Figure 2 shows a pie chart of the gene ontology terms that were enriched.

**Table 1:** The number of differentially expressed genes across different groups for absolute fold changes of 1.2, 1.5 and 2. All counted genes are significant at p-value < 0.05. Cluster A_st or Cluster B_st: individuals staying in the respective cluster A or B throughout the dietary intervention. Comparisons 1–5 are illustrated in Fig. 3.

	|FC| ≥ 1.2	|FC| ≥ 1.5	|FC| ≥ 2
Cluster A - Cluster B	1343	150	28
Comparison 1	1286	178	33
Comparison 2	282	24	1
Comparison 3	897	136	25
Comparison 4	669	75	16
Comparison 5	660	49	4
Cluster A_st - Cluster B_st	2838	460	97

A deeper look at the differentially expressed genes in the context of biological pathways (using PathVisio^35^ for pathway overrepresentation analysis) also revealed several metabolic pathways being differentially affected between the clusters. It appears that the overall cellular metabolism in Cluster A is decreased across the board; in the pathway overrepresentation results, the majority of the genes in the electron transport chain, fatty acid biosynthesis, fatty acid beta oxidation, glycolysis, and citric acid pathways are expressed lower (at least with a fold change of 1.2) in Cluster A compared to Cluster B. However, these are to be expected due to the way the ANF algorithm constructed the clusters based on the 291 metabolic flexibility-, and thus metabolism-related genes. On the other hand, we observed that pathways associated with the immune system and tissue restructuring were expressed higher in Cluster A compared to Cluster B. In particular, Complement C6 and C7, inflammatory interleukins IL1B and IL8, and matrix metalloproteinases MMP2 and MMP9 were found to be expressed higher in Cluster A. The differential expression analysis results, gene ontology enrichment results and pathway overrepresentation results are provided as Supplementary files 4–6 respectively.

### Cluster membership patterns

#### Cluster membership changers

**Figure 3:**
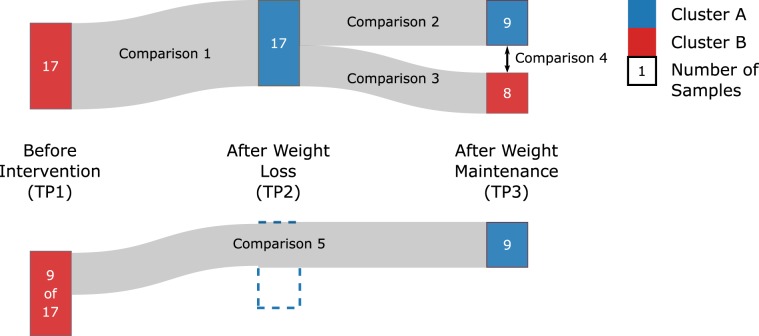
Illustration of the 17 subjects which switched from Cluster B to Cluster A during weight loss, after which 8 of the subjects reverted to Cluster B. The five comparisons used to break down and analyse the changes in the gene expression are also labelled.

Figure 1B shows the number of individuals in each cluster membership pattern found. We observed that a total of 17 individuals change their cluster membership from Cluster B to Cluster A when moving from time point 1 (TP1) to time point 2 (TP2); 9 of these individuals then stay in Cluster A until time point 3 (TP3), while the remaining 8 revert to their original cluster. We further analysed this pattern in the following comparisons (illustrated in Fig. 3; numbers of differentially expressed genes provided in Table 1).

##### Comparison 1

We first performed a paired differential expression analysis of all 17 individuals of this pattern between TP2 and TP1 to see the first changes that occurred when the individuals changed their cluster membership. A total of 1,286 differentially expressed genes were found in this comparison at absolute fold change equal to or greater than 1.2 and p-value less than 0.5. Gene ontology enrichment of these showed a similar pattern to the gene ontology enrichment when comparing the two clusters as a whole – the processes were broadly categorised into metabolic, immune/inflammatory and tissue morphology. This is understandable as these changes would be the reason for these 17 individuals being clustered in Cluster A at TP2 as opposed to Cluster B at TP1. Similarly, the pathway enrichment results also follow the same pattern as that of the whole cluster comparison. Genes in metabolic processes such as fatty acid beta oxidation, electron transport chain complexes, glycolysis, lipogenesis, gluconeogenesis and amino acid metabolism were down regulated at TP2 Cluster A compared to when these individuals were in Cluster B at TP1. Genes involved in inflammation, on the other hand, were found to be upregulated in the same pattern as the whole cluster comparison.

##### Comparison 2

We performed a paired differential expression analysis between TP3 and TP2 for the 9 individuals that retained their new cluster to see what changes occurred even when the cluster membership did not change. In this comparison, we found 282 differentially expressed genes. Considering that this comparison is between the 9 individuals which stay in Cluster A at both TP2 and TP3, the low number is expected. Consequently, the gene ontology enrichment and pathway overrepresentation analysis also do not show any major differences between these samples. Of the minor differences found, peroxisome proliferator activated receptor gamma (PPAR*γ*), fatty acid synthase (FASN) and stearoyl-CoA desaturase (SCD) were found to be upregulated in these individuals at TP3 compared to TP2. This is interesting, especially in the light of our previous studies covering the role of these proteins in metabolic flexibility. However, the lack of strong results from the two analyses overall make it difficult to infer the effects of their upregulation concretely.

##### Comparison 3

We performed a paired differential expression analysis between TP3 and TP2 for the 8 individuals that changed back to the original cluster to see what changes occurred when these individuals returned to their original cluster. In this comparison, we found 897 differentially expressed genes. The gene ontology enrichment analysis for these genes showed that these were also tied to cellular metabolic processes, with two immune system related terms also enriched. Compared to the previous two parts, the individuals in this analysis showed a reversed trend where the immune system related pathways were being downregulated at TP3 compared to TP2. The human complement system pathway and toll-like receptor (TLR) associated pathways showed downregulation of respective components. The matrix metalloproteinase MMP9 was also strongly downregulated. On the other hand, upregulated genes showed an upregulation of metabolism related processes across the board with lipid metabolism and biosynthesis showing the highest z-scores in pathway overrepresentation. Sterol regulatory element-binding protein (SREBP) signalling also showed strong upregulation compared to both TP2 in these individuals, as well as the previous two parts. Also in contrast to the previous two parts, as well as the whole cluster differential expression, was the upregulation of leptin (LEP) and downregulation of leptin receptor (LEPR). In addition, glycolysis and gluconeogenesis enzymes were mostly found to be slightly upregulated.

##### Comparison 4

We performed a differential expression analysis between the 17 individuals at TP3 to see which changes were retained by these individuals by the final time point. In this comparison, a total of 669 genes were found to be differentially expressed genes between 17 individuals, 8 at TP3 in Cluster B and 9 at TP3 in Cluster A. The gene ontology enrichment showed a limited number of terms, all associated with cellular metabolism. Pathway overrepresentation showed genes from several immune system related processes, such as interferon signalling and interleukin signalling to be downregulated. Metabolism associated processes, on the other hand, were found to be upregulated. Overall, the results of this analysis mirror those of the previous part.

##### Comparison 5

We performed a paired differential expression analysis between the 9 individuals at TP3 and TP1 to see the changes in metabolic processes, if any, between their old cluster at TP1 and their new cluster at TP3. In this comparison, we observed a total of 660 differentially expressed genes, with the gene ontology enrichment and pathway overrepresentation results following the same pattern as that in Comparison 1. The reduction in the number of differentially expressed genes only affected the intensity of the gene expression and the z-score in the overrepresentation analysis. Due to this, the pathways results are difficult to interpret as considerable parts of many pathways have varied expression patterns between the individuals and high p-values.

#### Cluster membership maintainers

We also observed that a number of individuals do not change their cluster membership at all and stay within Cluster A (4 individuals) or Cluster B (8 individuals) throughout the study. The differential gene expression analysis between individuals staying in Cluster A and individuals staying in Cluster B yields the largest number of differentially expressed genes, at 2,838, compared to the previous analyses. Gene ontology enrichment analysis showed a combination of metabolic, inflammatory and tissue morphology terms that were enriched. The pathway overrepresentation showed that individuals staying in Cluster A persistently had a lower overall expression of cellular metabolism with various enzymes across lipid metabolism, SREBP signalling, mitochondria electron transport chain, TCA cycle, and glycolysis being downregulated compared to individuals in Cluster B. Immune system processes, such as components of the human complement system and interleukin signalling were found to be upregulated in Cluster A. In addition, matrix metalloproteinases MMP2, MMP7 and MMP9 were also found to be upregulated in Cluster A.

The differential expression analyses output, gene ontology enrichments output, as well as the pathway overrepresentation analysis output for the aforementioned results are provided as Supplementary files 4–6 respectively.

## Discussion

### Lowered cellular metabolism

In the study presented in this article, we have analysed transcriptomics and proteomics expression data of the subcutaneous adipose tissue to stratify the individuals based on the expression of their genes and proteins involved in cellular metabolism, to construct metabolic profiles based on the roles of these genes and proteins in cellular metabolic flexibility. We clustered the samples of the metabolic flexibility related transcriptomics and proteomics data to generate two clusters, A and B, which showed marked difference in their whole gene expression profile. The clustering pattern generated by the ANF algorithm showed an overall lowered expression of metabolism related genes in Cluster A compared to Cluster B. Interestingly, a larger number of individuals started in Cluster B, changed their metabolic profile in response to caloric restriction and joined Cluster A, and then about half of them reverted to their original profile. This is likely related to the fact that the individuals in the study were overweight before dietary intervention, and their response to the caloric restriction is reflected in them changing clusters. However, it is interesting that several individuals maintained their new lowered metabolic gene expression beyond caloric restriction into weight maintenance, although, given the time period between the data collection points (4 weeks), it is difficult to assess the long term impact and/or changes in metabolic profile. The phenotypic/clinical measurements did not show any major difference between the two clusters, apart from the significant anthropometric measurement of sex, height, BMI, fat mass, fat free mass, waist size and hip circumference. These are explained by the clustering pattern that we observe where there is a disproportionate number of before weight loss samples in Cluster B compared to Cluster A, and vice versa after weight loss. As most of these anthropometric measurements are related, and are partially interrelated, their simultaneous significance is not surprising in light of the clustering pattern. Consequently, we also did not see any difference in the weight maintenance score (s-values) of the two clusters either, indicating that the impact of the metabolic profiles built on the metabolic flexibility related genes is much subtler than we had expected. However, we did see a significant difference (p-value = ~0.03) in the s-values for the nine subjects which changed their profile over the course of the dietary intervention from Cluster B (TP1) to Cluster A (TP2), when compared to the s-values of the eight subjects which stayed in Cluster B throughout the dietary intervention.

### Increased inflammatory response

Comparing the samples of the two clusters as a whole shows that apart from the difference in cellular metabolism, expected as the clustering was based on the 291 cellular metabolism associated genes, the two clusters also show a difference in immune related processes in the adipose tissue expression data. Interestingly, the immune system processes, such as interferon signalling, toll-like receptor signalling and interleukin signalling were expressed higher in Cluster A compared to Cluster B, while the metabolic processes were being downregulated in Cluster A compared to Cluster B. The immune system response in terms of inflammation is associated with obesity^38–42^, as such we find it interesting that immune processes were being upregulated in Cluster A, compared to Cluster B, especially since there is a larger proportion of TP2 samples in Cluster A (TP2 is the time point immediately after weight loss in the data). It is possible that, because of weight loss, the adipocytes in the adipose tissue shrink in size, and thus the adipose tissue samples post weight loss contain a larger proportion of immune cells as compared to adipocytes. However, this is unlikely, as the differential expression analysis between Cluster A at TP3 and Cluster B at TP3 shows a lower immune system associated gene expression in Cluster B samples compared to those of Cluster A. This result suggests that the immune system expression is not tied to the relative proportion of cells in the collected samples; otherwise, the immune system related gene expression would not have been significantly different between the two clusters at TP3. However, this is purely deductive reasoning, and a study on the histological/morphological data of the subcutaneous adipose tissue during weight loss would be required to conclusively (in)validate this perspective.

### Implication in the context of metabolic flexibility

Some recent studies in mice have shown an upregulation of macrophage related immune activity in the mouse adipose tissue after weight loss^43,44^. Indeed, a previous report from the Yoyo study data also found leukocyte integrin gene activity in relation to weight regain post weight loss using a set of extracellular matrix related genes^45^. These studies, collectively, indicate that the continued immune activity after weight loss may explain and/or contribute to the variation seen in the weight regain after weight loss. Other studies cite persistent low-grade inflammation as a cause for chronic non-communicable diseases^46–48^. It has also been shown that localised high expression of tumour necrosis factor (TNF) and interleukin-6 (IL-6) are associated with obesity induced insulin insensitivity^49^. Given that we know that metabolic inflexibility is associated with chronic illnesses such as type 2 diabetes mellitus, cardiovascular diseases and metabolic syndrome^2–4,50^, it is possible that there exists a link through the persistent low-grade inflammation. This is certainly possible as in one of our previous studies^21^, we showed how PPAR*γ* plays a role in regulating cellular metabolic flexibility, while it is also known that PPAR*γ* has connections to the immune system^51–53^.

As mentioned earlier, we see an increase in immune response after weight loss and/or weight maintenance in the majority of the individuals of the data analysed. This observation complicates the interpretation as weight loss is expected to improve or restore metabolic flexibility, given the reduction of obesity. Furthermore, based on how the ANF algorithm produces the clustering, we expect to find at least the metabolic flexibility genes to be different in the two clusters, introducing a minor, yet inherent bias. In the data we analysed in this study, we did not have the time scale resolution required to decipher more concrete links between metabolic flexibility, adipose tissue metabolism and the immune response. However, our study still provides hints towards this interaction. From a strictly statistical perspective, a considerable portion of the differential expression results in this study become insignificant when adjusted for multiple correction. Although this insignificance has little impact of enrichment analyses and their results, it still shows that much work needs to be done in order to untangle these cellular interactions and their effects. As such, the interplay of these systems, at the very least in the adipose tissue, needs to be further elucidated in more targeted studies as an important step towards combatting and containing obesity and associated co-morbidities. Although we cannot comment on the technology that might be required for accurately measuring the rate of change of cellular metabolism, perhaps it might be possible to indirectly infer such a rate using cell line experiments. In addition, given that the data utilised in this article was a dietary intervention dataset, it would be interesting to analyse a dataset comparing obese and lean individuals on the same data types and parameters to observe if any combination of parameters stand out as phenotypically defining metabolic inflexibility.

## Conclusions

Cellular metabolism and metabolic flexibility have been shown to be associated with obesity and the development of chronic illnesses such as type 2 diabetes mellitus and metabolic syndrome. In our study, we clustered gene expression samples from a weight loss study into two clusters, based on 291 genes associated with cellular metabolic flexibility. Our analyses showed that the majority of the individuals had their metabolism associated genes downregulated after weight loss and weight maintenance, but also had an upregulation of immune system associated genes. A higher expression of the immune system has previously been associated with the impairment of metabolism post-weight loss in mice. Our study suggests a similar pattern in the human adipose tissue, opening the way for targeted studies to elucidate the interactions of metabolism, metabolic flexibility and the immune system in the context of chronic diseases. Furthermore, it was observed that individuals which had changed their metabolic profiles in response to caloric restriction (as reflected by the change in Cluster membership from B to A) had a significant retention of lost weight compared to individuals which had not changed their cluster membership (i.e., remained in Cluster B).

## Supplementary information

Supplementary file 1.

Supplementary file 2.

Supplementary file 3.

Supplementary file 4.

Supplementary file 5.

Supplementary file 6.

## Data Availability

The expression data analysed in the study is available at gene expression omnibus (GEO) under accession ID: GSE77962. The proteomics data and clinical/phenotypic measurements are freely available from the authors upon request.
