# Supplementary figures and images for: Stratifying cellular metabolism during weight loss: an interplay of metabolism, metabolic flexibility and inflammation
Source: Sci Rep. 2020 Feb 3;10:1651. doi: 10.1038/s41598-020-58358-z (PMC6997359; doi:10.1038/s41598-020-58358-z)

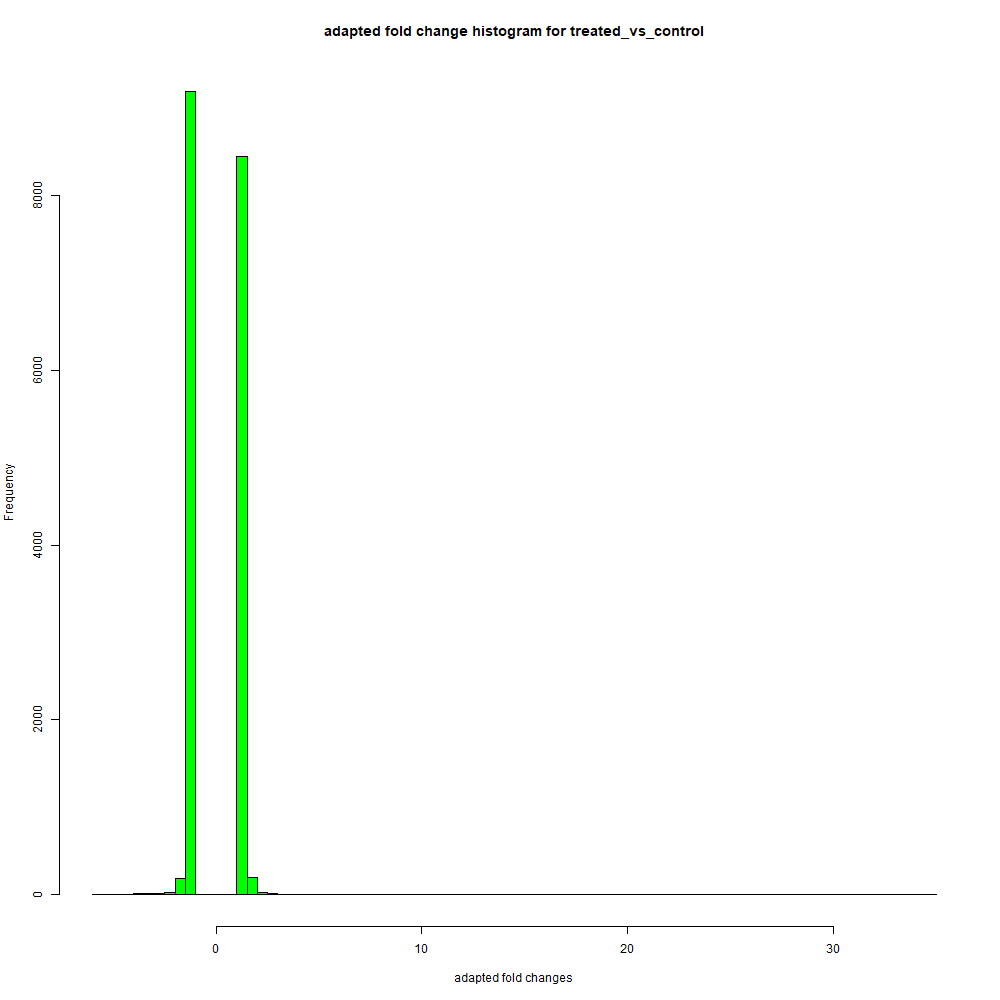

Supplement: Supplementary file 4 [file 41598_2020_58358_MOESM4_ESM.zip › CA_st - CB_st/FC_hist_treated_vs_control.png]

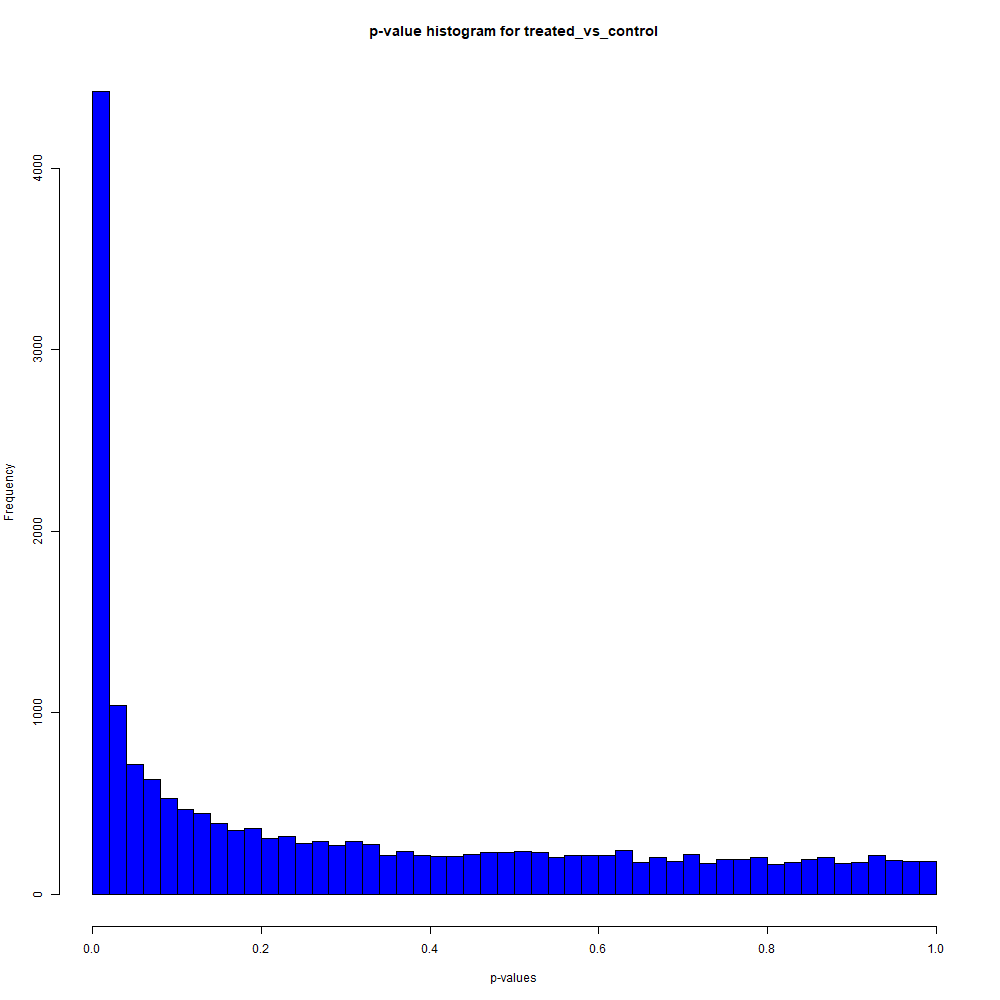

Supplement: Supplementary file 4 [file 41598_2020_58358_MOESM4_ESM.zip › CA_st - CB_st/pvalue_hist_treated_vs_control.png]

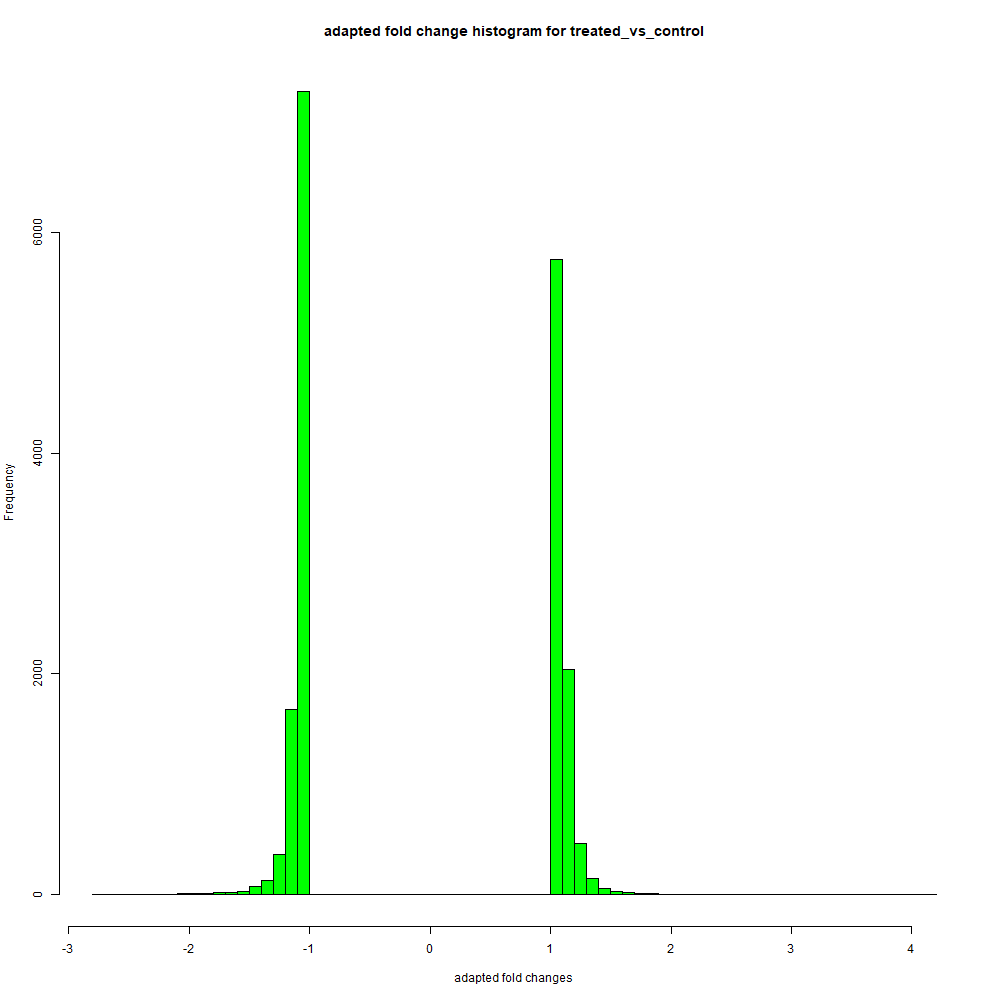

Supplement: Supplementary file 4 [file 41598_2020_58358_MOESM4_ESM.zip › Cluster A - Cluster B/FC_hist_treated_vs_control.png]

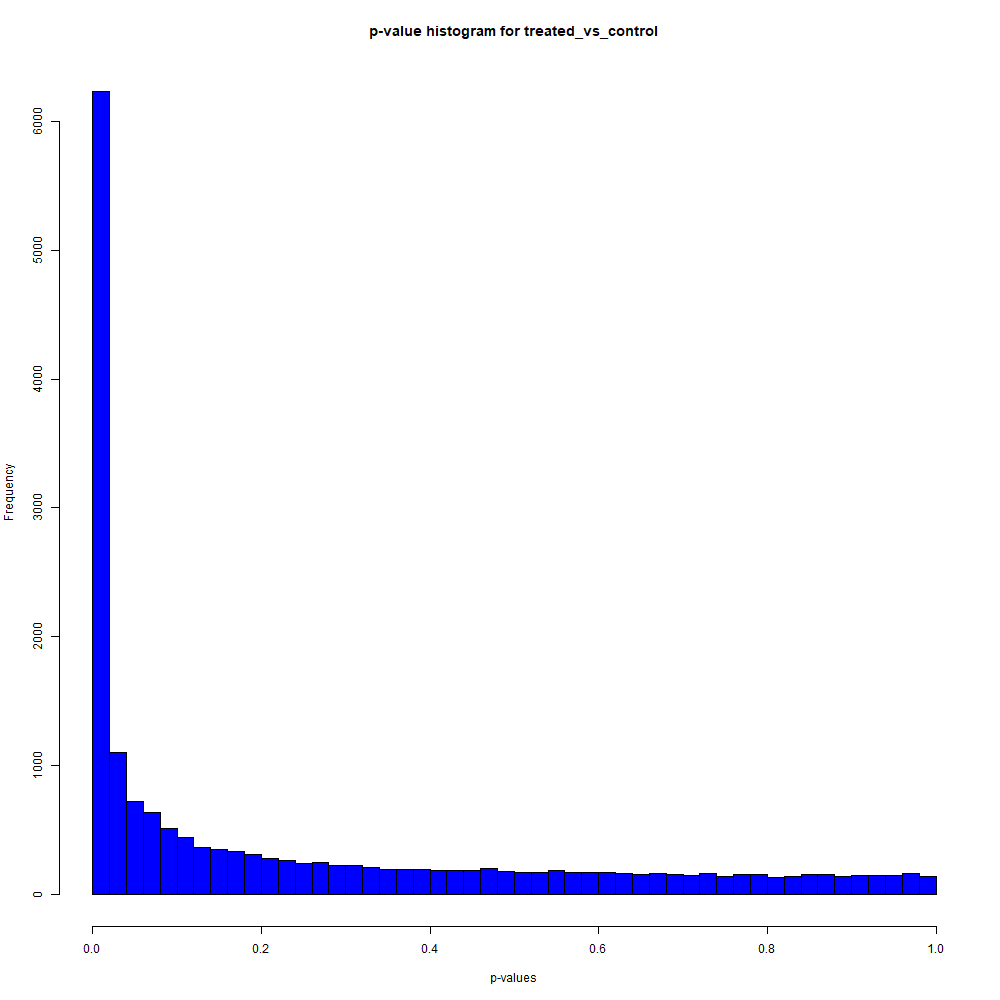

Supplement: Supplementary file 4 [file 41598_2020_58358_MOESM4_ESM.zip › Cluster A - Cluster B/pvalue_hist_treated_vs_control.png]

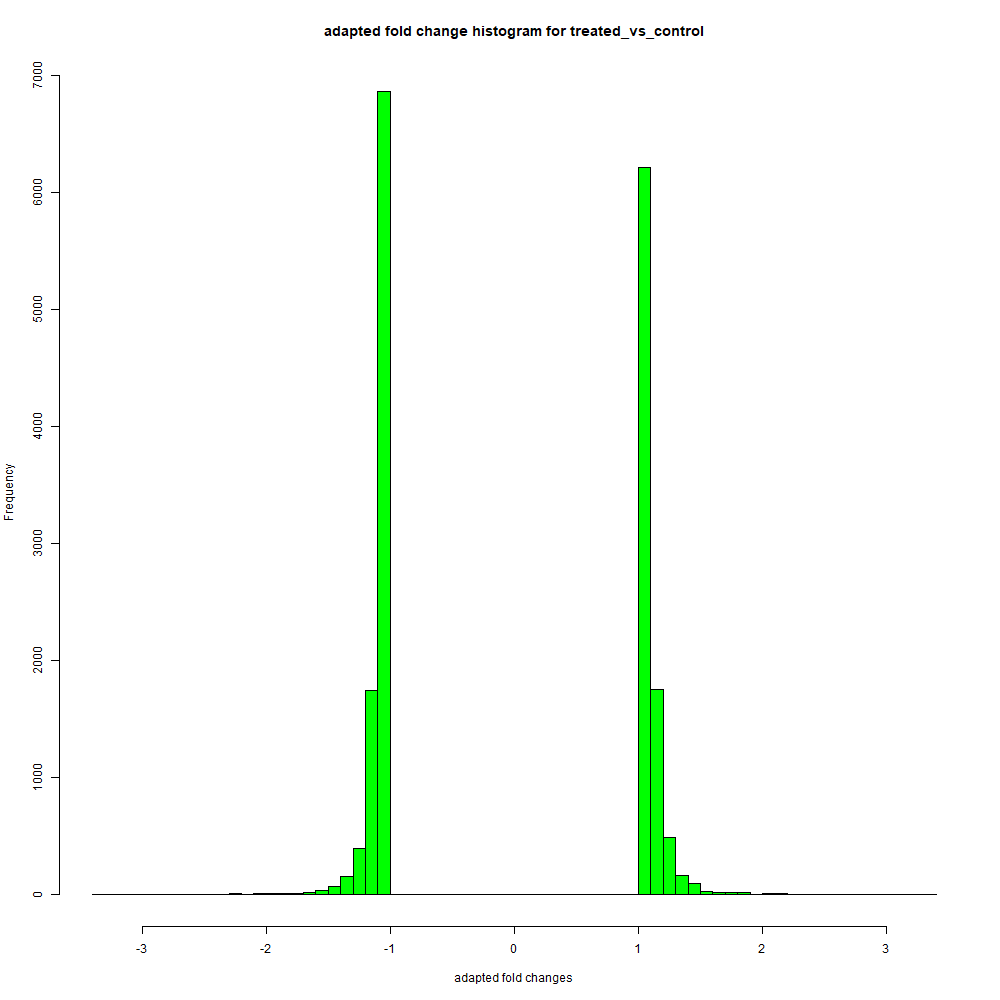

Supplement: Supplementary file 4 [file 41598_2020_58358_MOESM4_ESM.zip › Comparison 1/FC_hist_treated_vs_control.png]

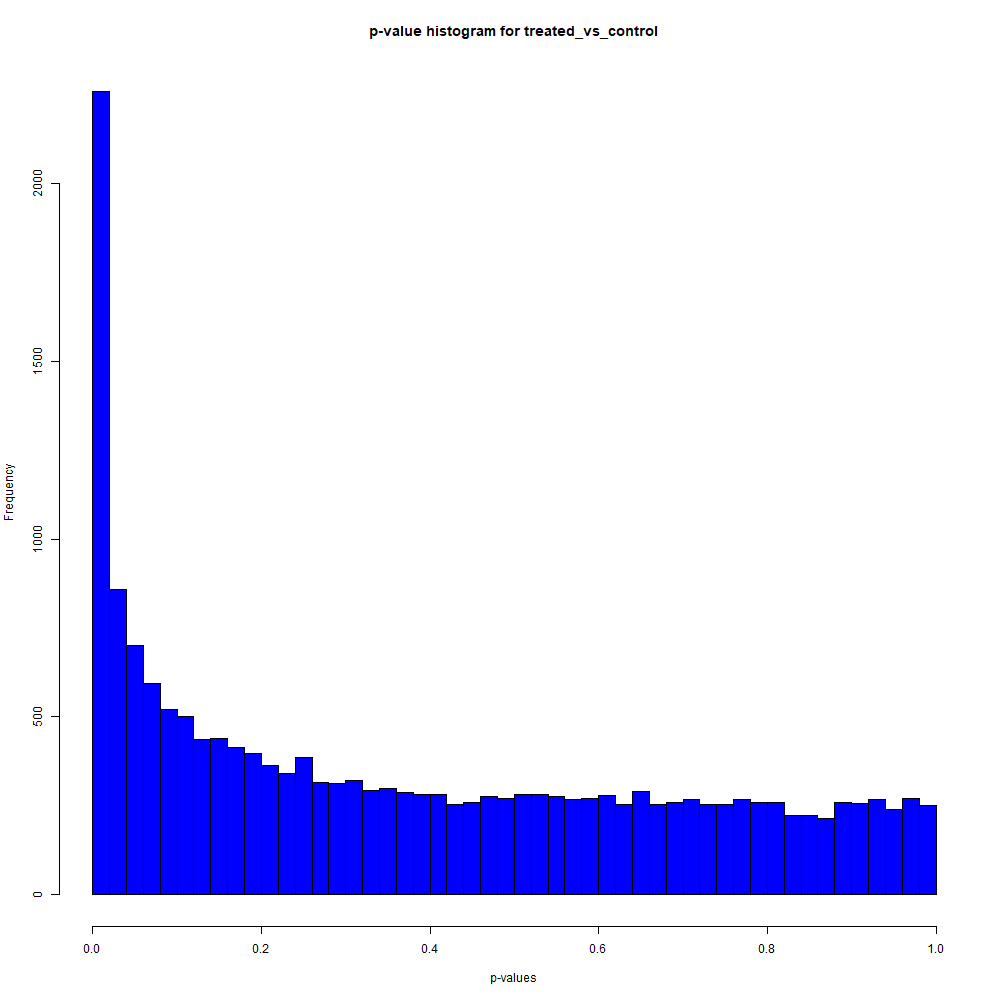

Supplement: Supplementary file 4 [file 41598_2020_58358_MOESM4_ESM.zip › Comparison 1/pvalue_hist_treated_vs_control.png]

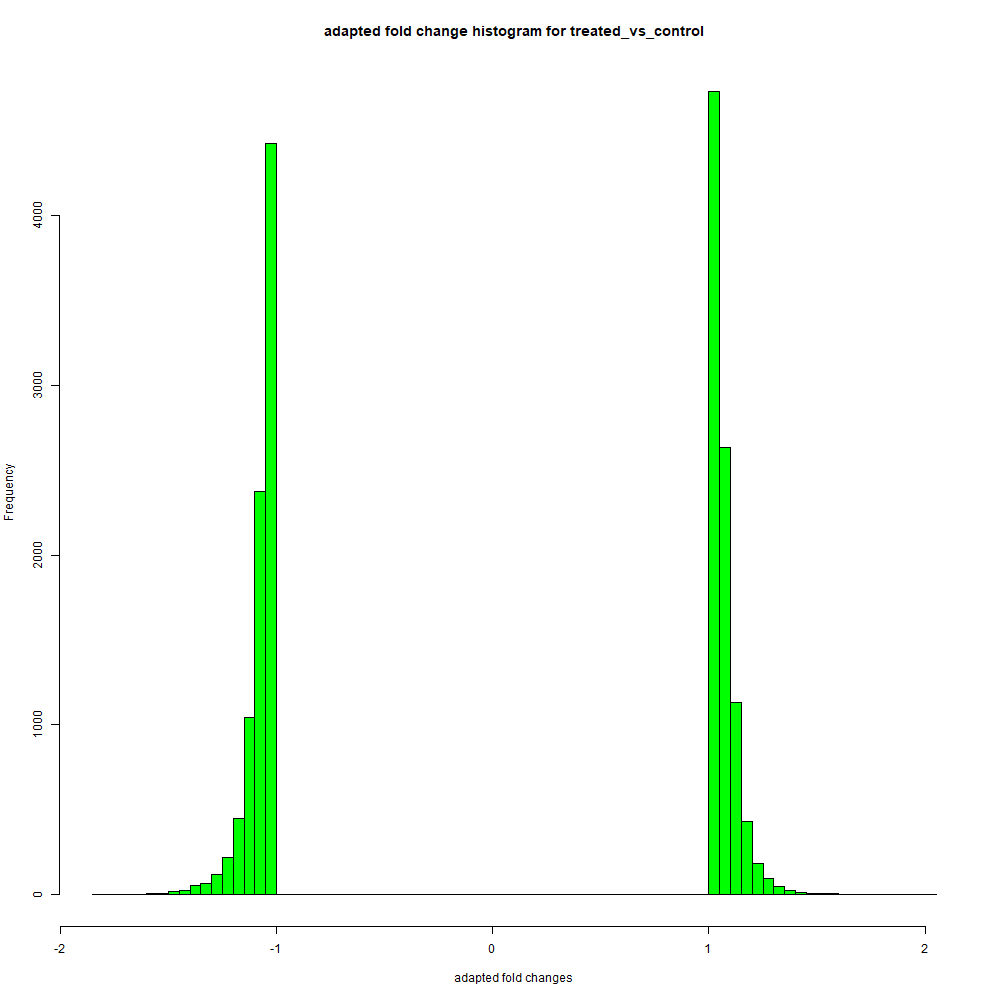

Supplement: Supplementary file 4 [file 41598_2020_58358_MOESM4_ESM.zip › Comparison 2/FC_hist_treated_vs_control.png]

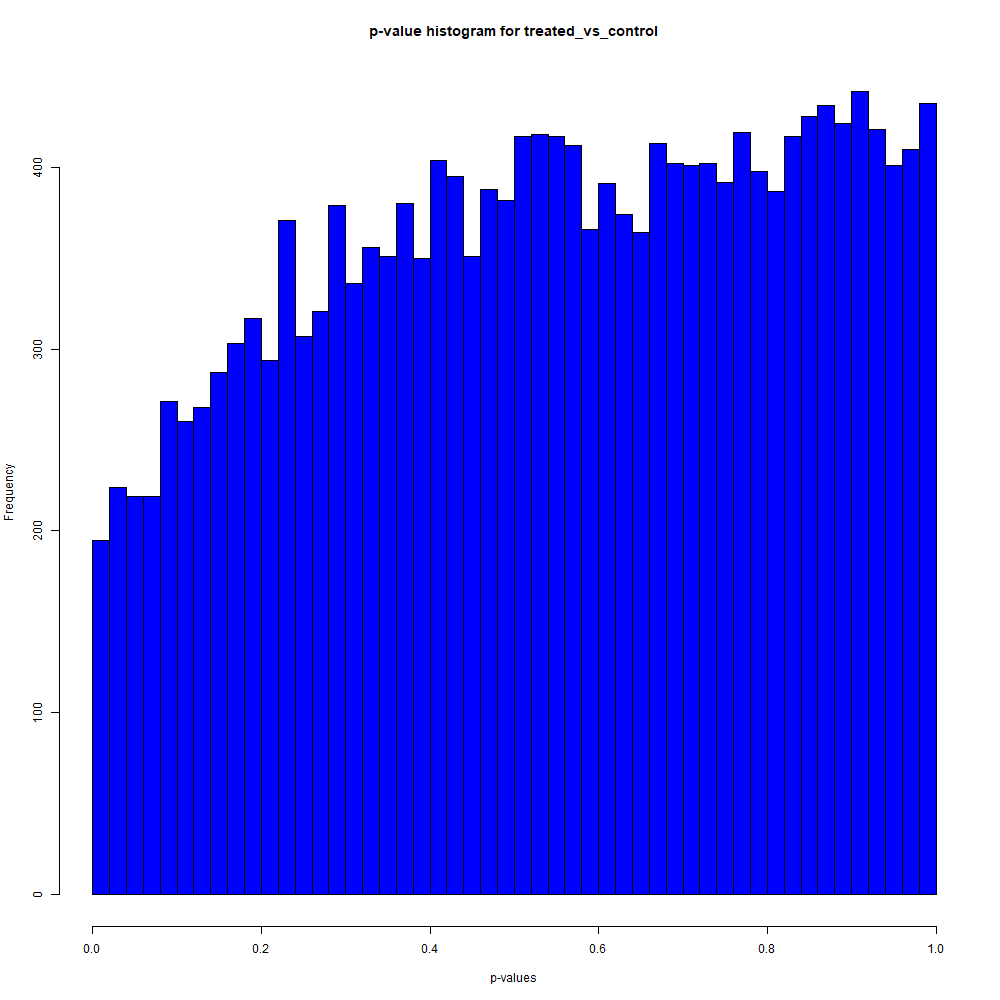

Supplement: Supplementary file 4 [file 41598_2020_58358_MOESM4_ESM.zip › Comparison 2/pvalue_hist_treated_vs_control.png]

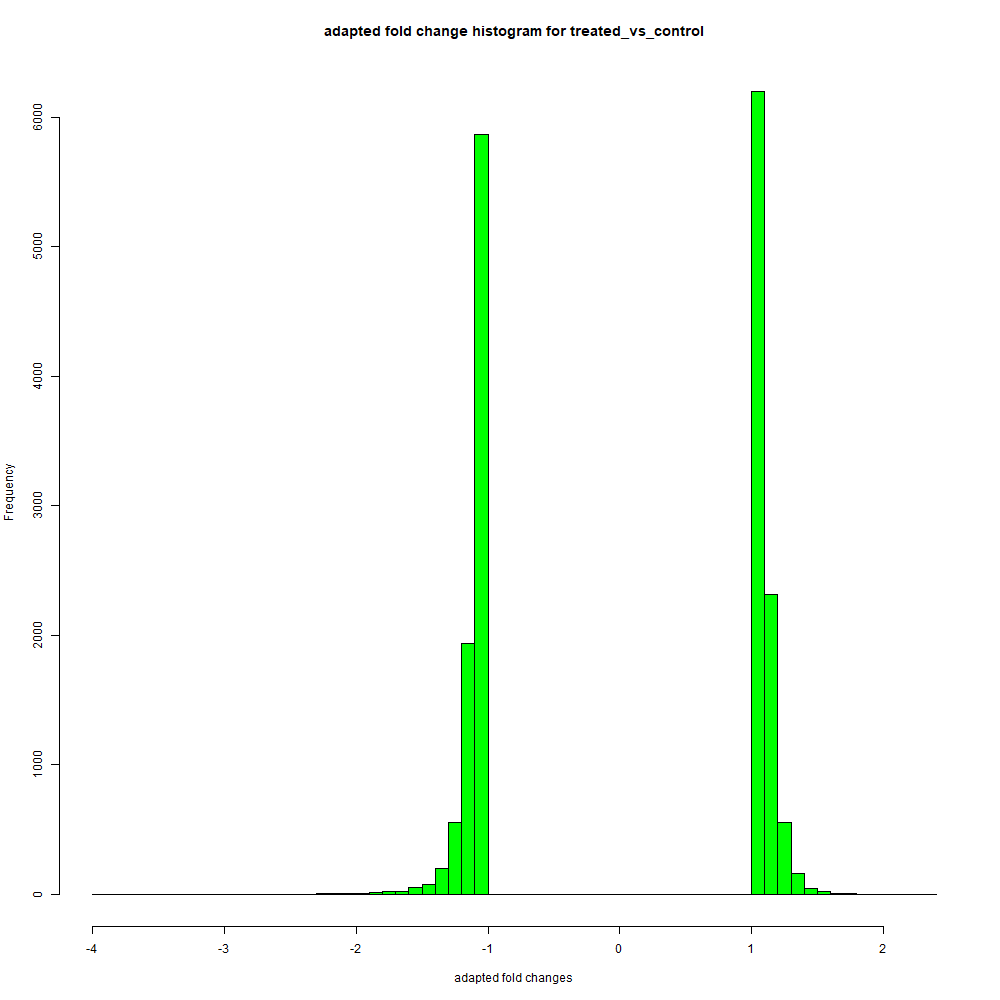

Supplement: Supplementary file 4 [file 41598_2020_58358_MOESM4_ESM.zip › Comparison 3/FC_hist_treated_vs_control.png]

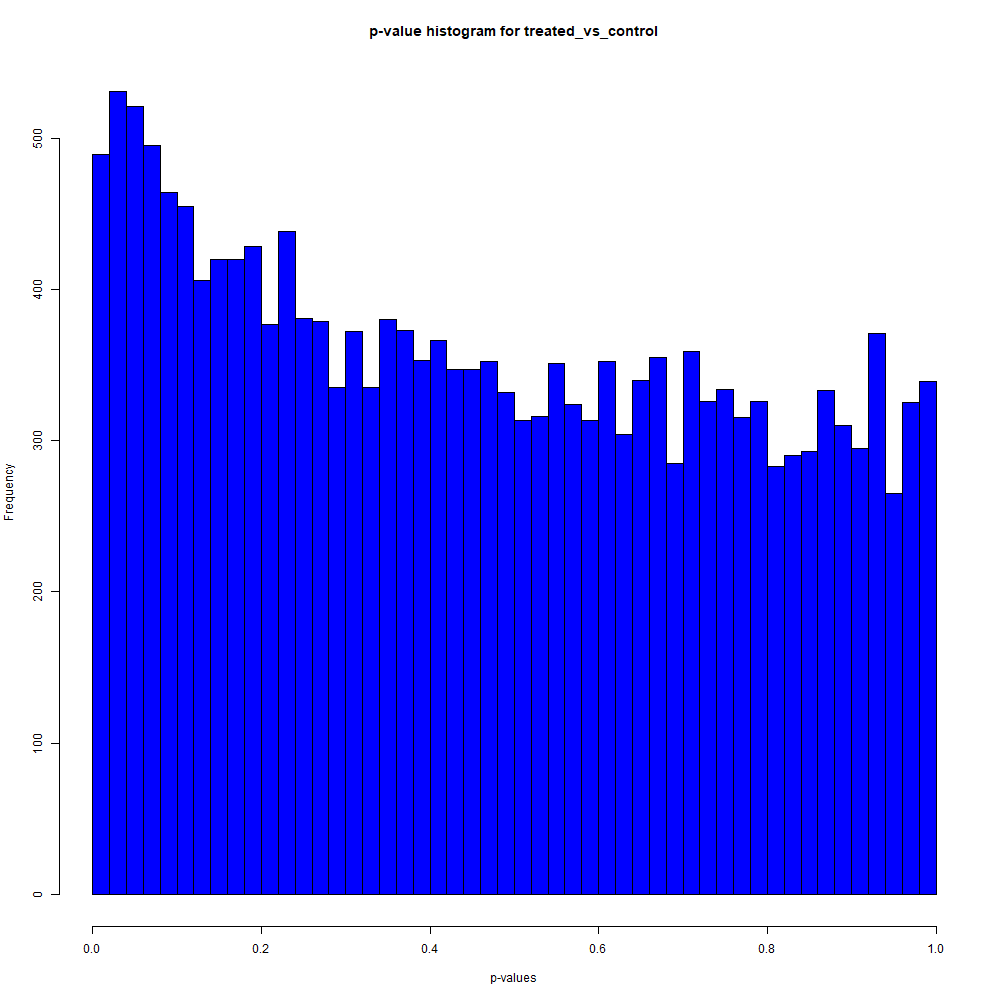

Supplement: Supplementary file 4 [file 41598_2020_58358_MOESM4_ESM.zip › Comparison 3/pvalue_hist_treated_vs_control.png]

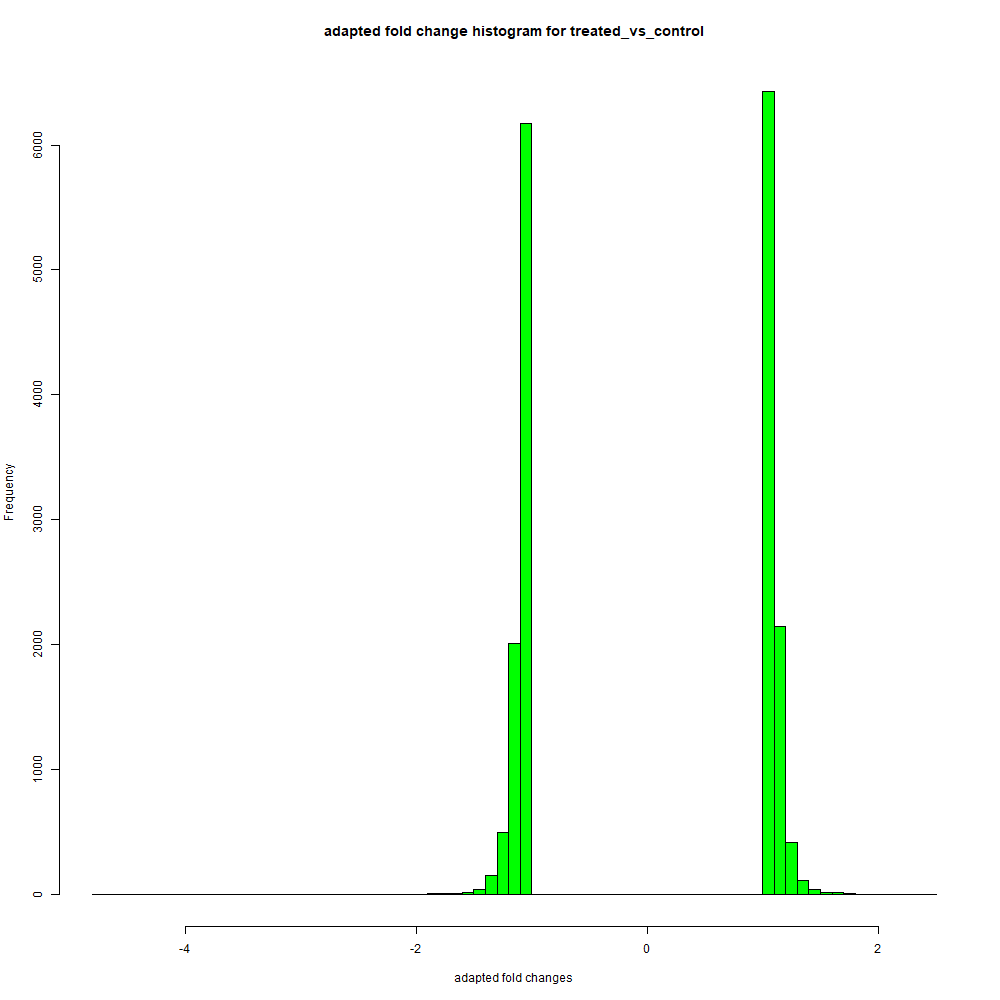

Supplement: Supplementary file 4 [file 41598_2020_58358_MOESM4_ESM.zip › Comparison 4/FC_hist_treated_vs_control.png]

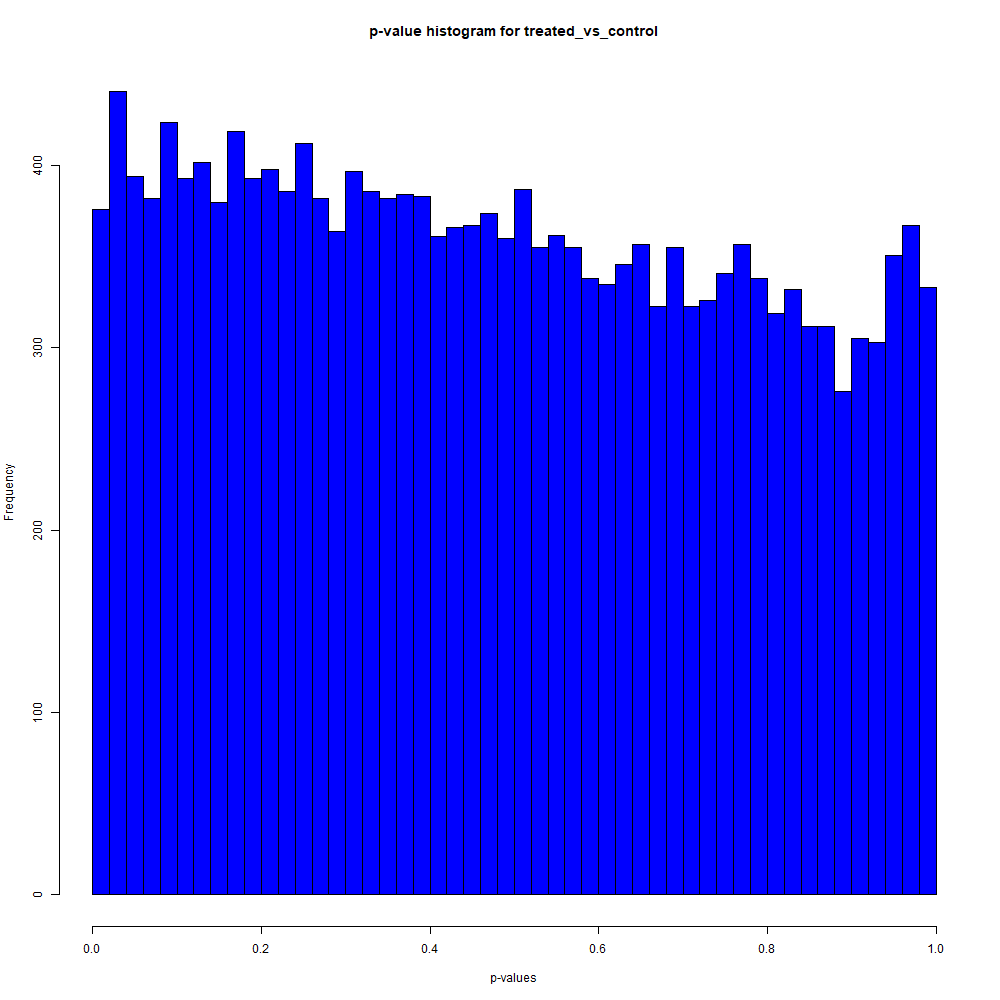

Supplement: Supplementary file 4 [file 41598_2020_58358_MOESM4_ESM.zip › Comparison 4/pvalue_hist_treated_vs_control.png]

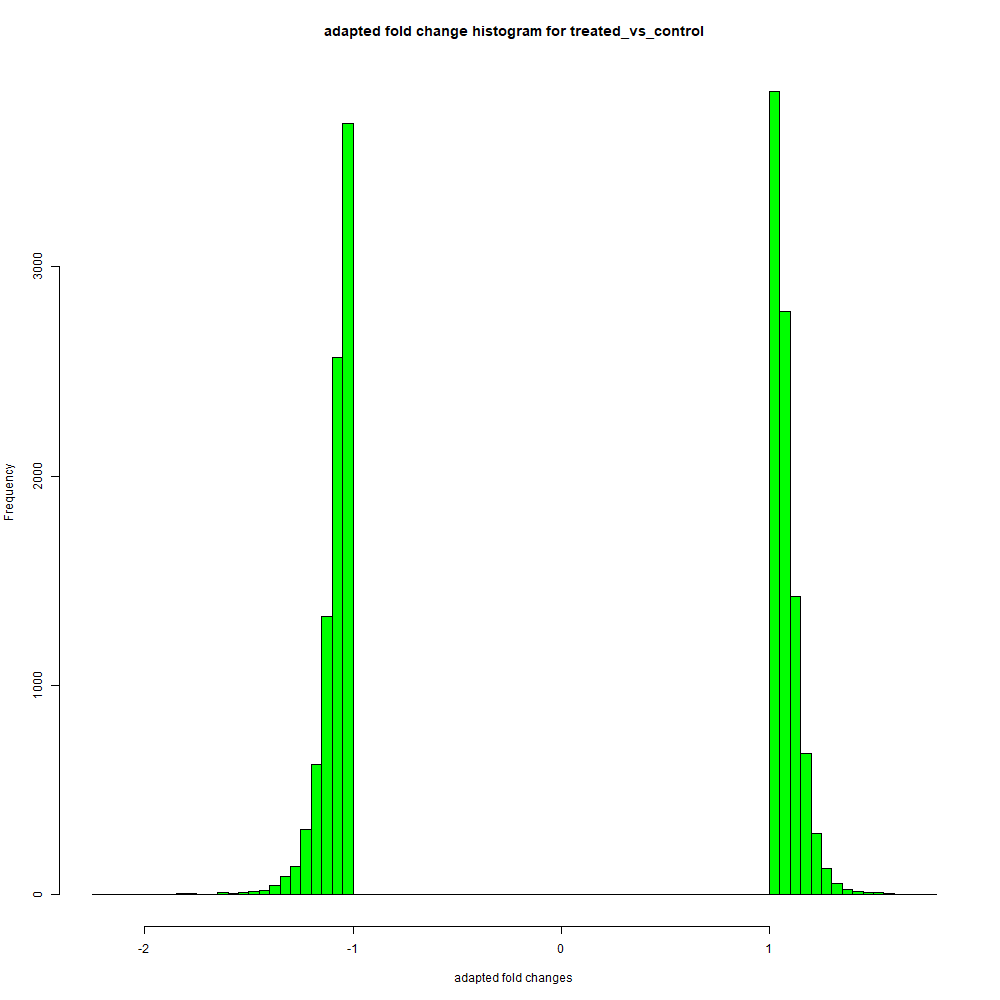

Supplement: Supplementary file 4 [file 41598_2020_58358_MOESM4_ESM.zip › Comparison 5/FC_hist_treated_vs_control.png]

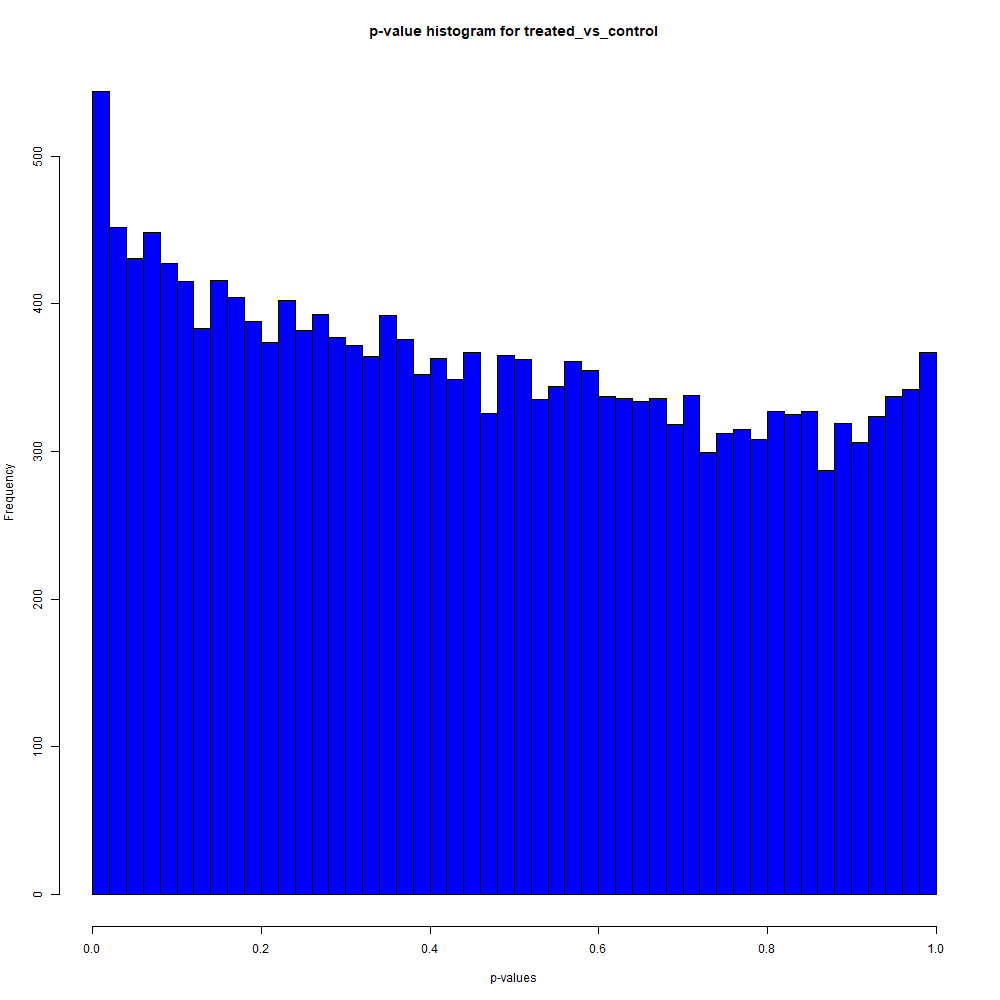

Supplement: Supplementary file 4 [file 41598_2020_58358_MOESM4_ESM.zip › Comparison 5/pvalue_hist_treated_vs_control.png]

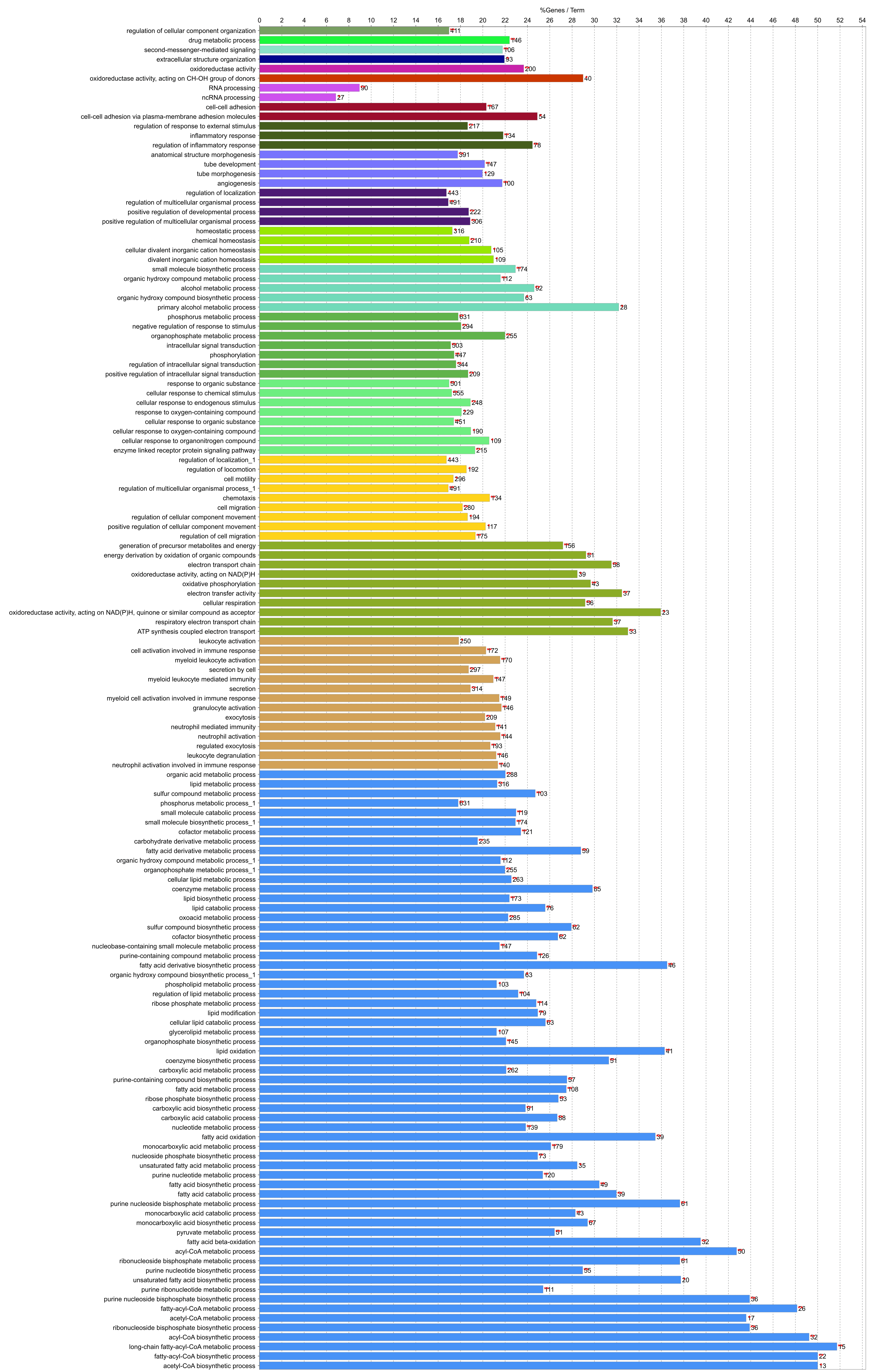

Supplement: Supplementary file 5 [file 41598_2020_58358_MOESM5_ESM.zip › CA_st - CB_st/BarChart-comp.png]

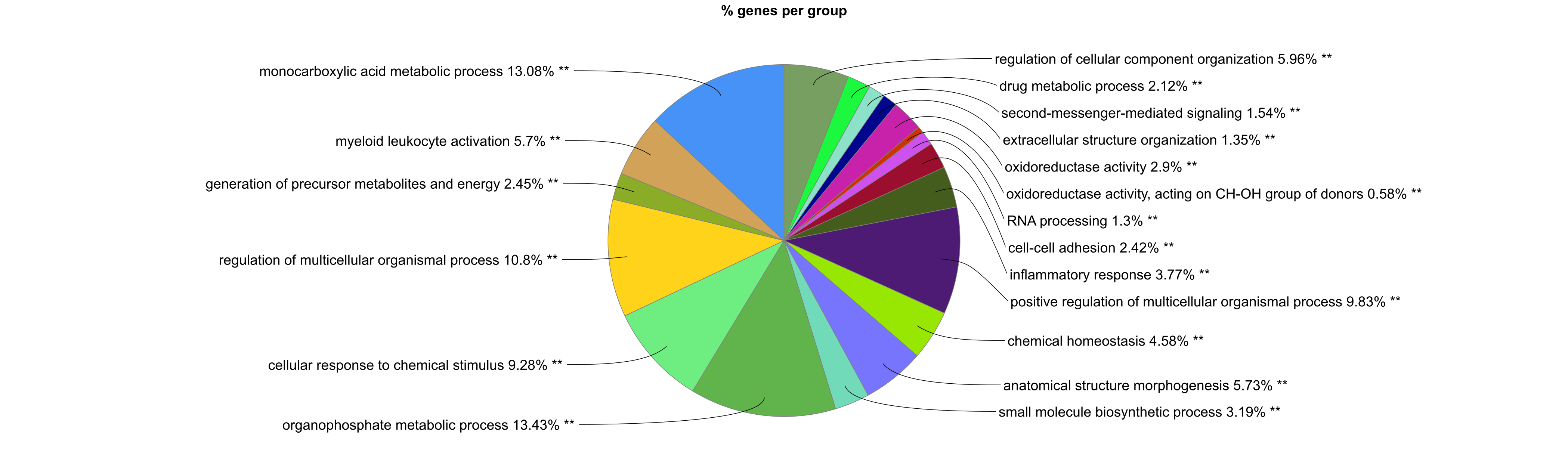

Supplement: Supplementary file 5 [file 41598_2020_58358_MOESM5_ESM.zip › CA_st - CB_st/PieChart_genes.png]

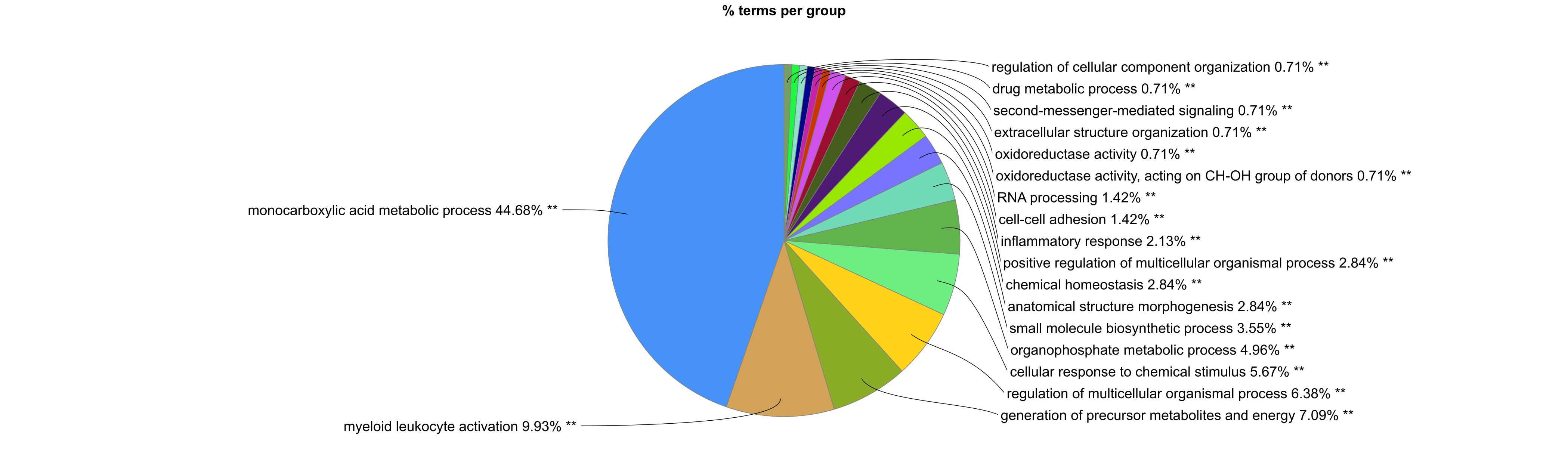

Supplement: Supplementary file 5 [file 41598_2020_58358_MOESM5_ESM.zip › CA_st - CB_st/PieChart_terms.png]

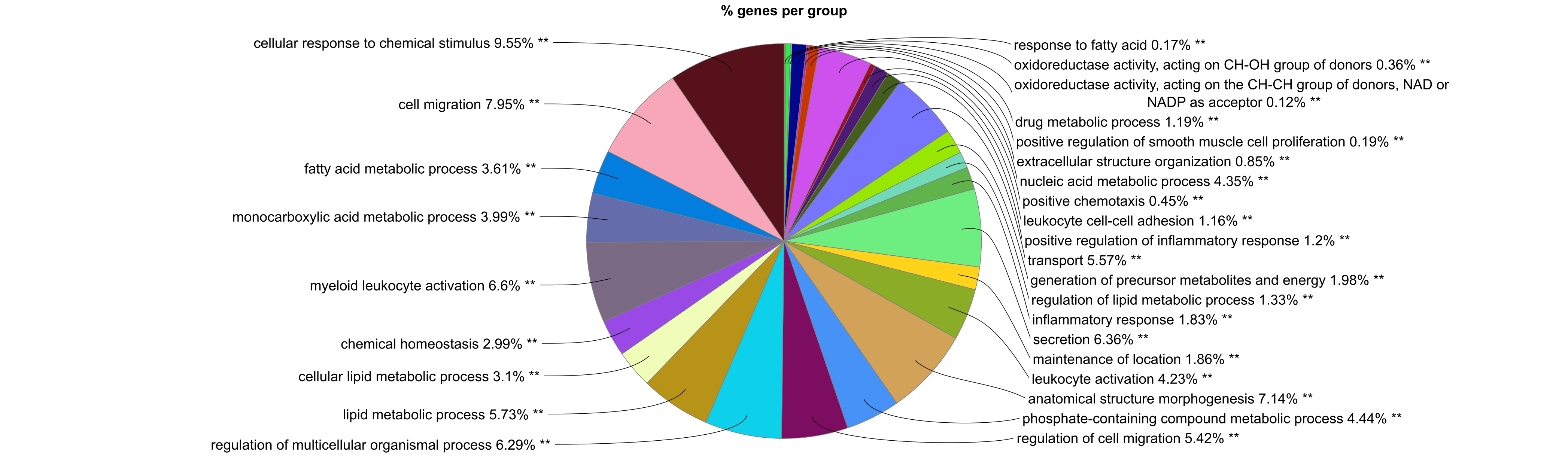

Supplement: Supplementary file 5 [file 41598_2020_58358_MOESM5_ESM.zip › Cluster A - Cluster B/PieChart_genes.PNG]

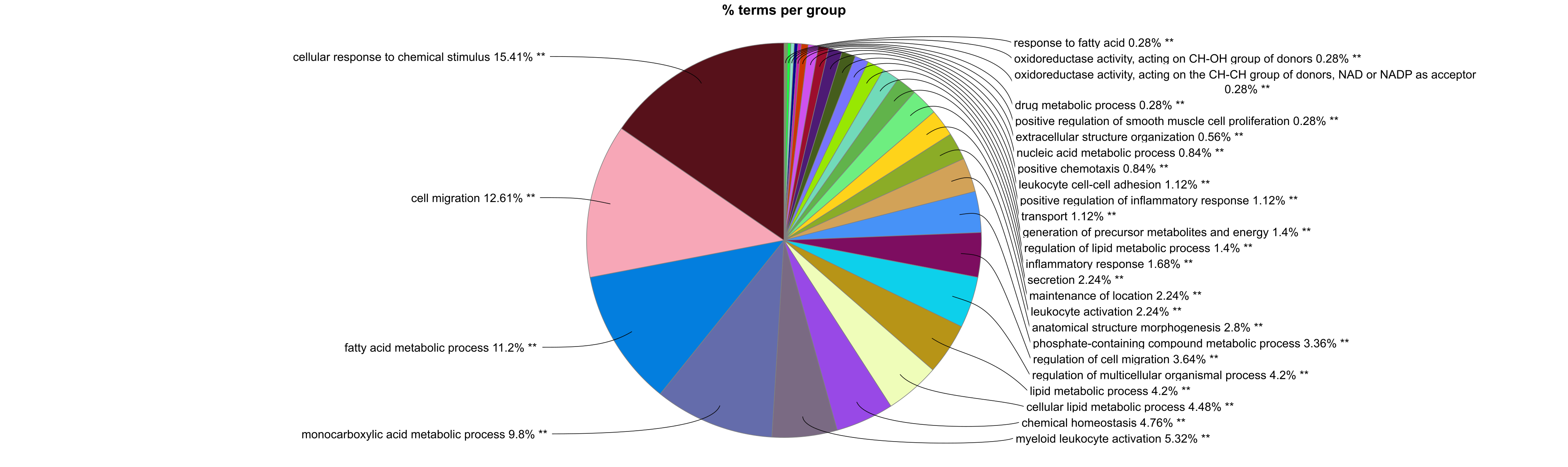

Supplement: Supplementary file 5 [file 41598_2020_58358_MOESM5_ESM.zip › Cluster A - Cluster B/PieChart_terms.PNG]

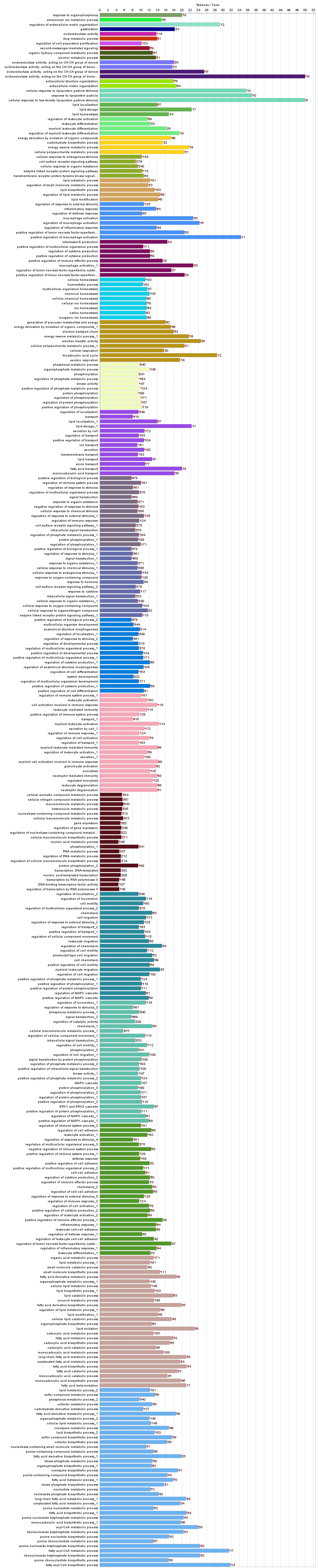

Supplement: Supplementary file 5 [file 41598_2020_58358_MOESM5_ESM.zip › Comparison 1/BarChart.png]

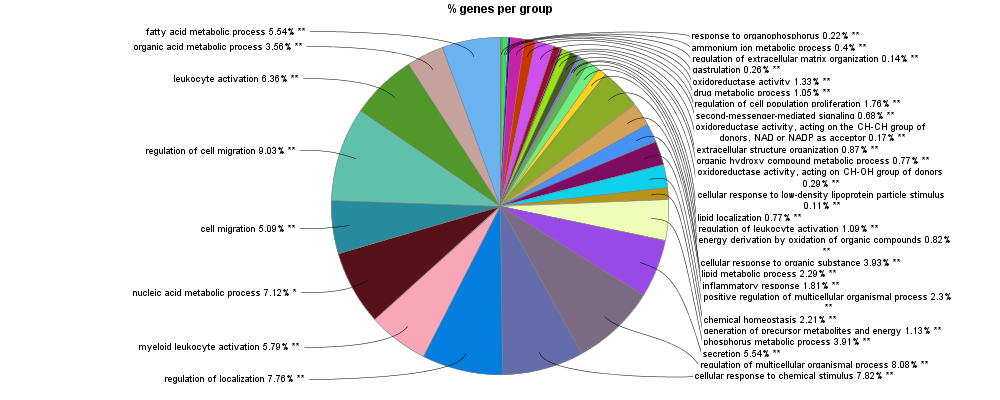

Supplement: Supplementary file 5 [file 41598_2020_58358_MOESM5_ESM.zip › Comparison 1/PieChart_genes.png]

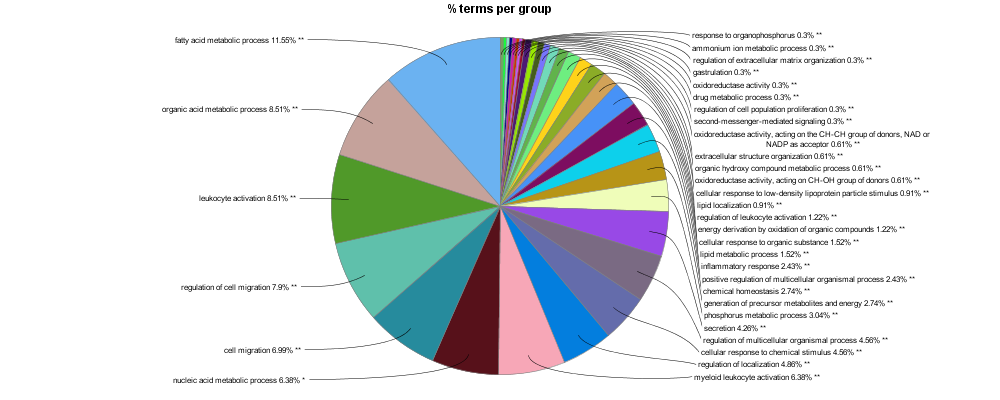

Supplement: Supplementary file 5 [file 41598_2020_58358_MOESM5_ESM.zip › Comparison 1/PieChart_terms.png]

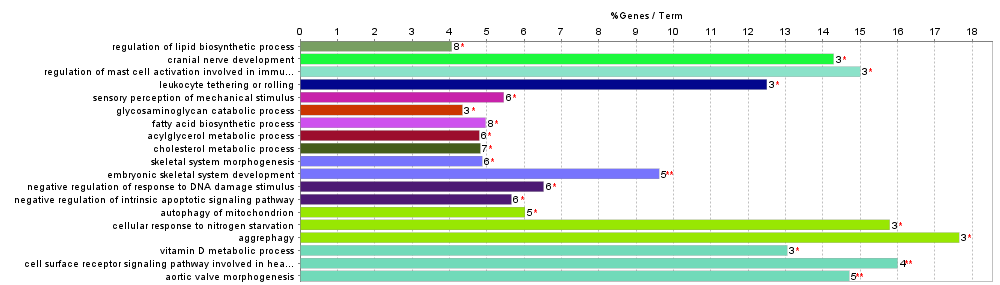

Supplement: Supplementary file 5 [file 41598_2020_58358_MOESM5_ESM.zip › Comparison 2/BarChart.png]

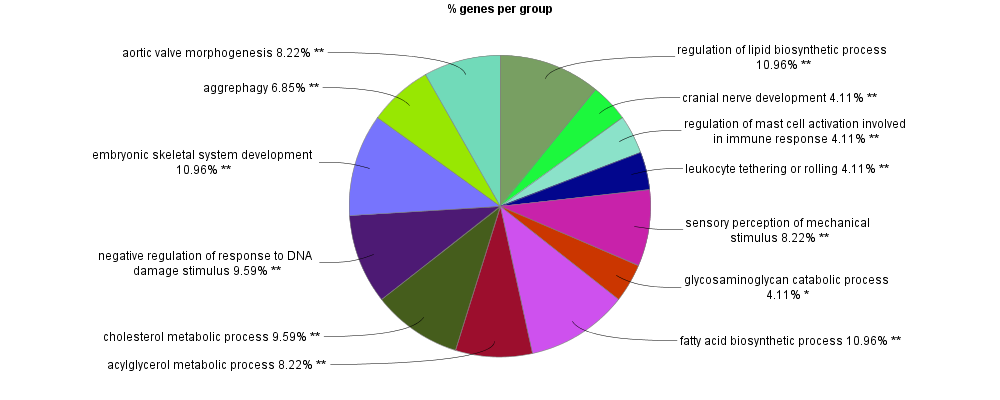

Supplement: Supplementary file 5 [file 41598_2020_58358_MOESM5_ESM.zip › Comparison 2/PieChart_genes.png]

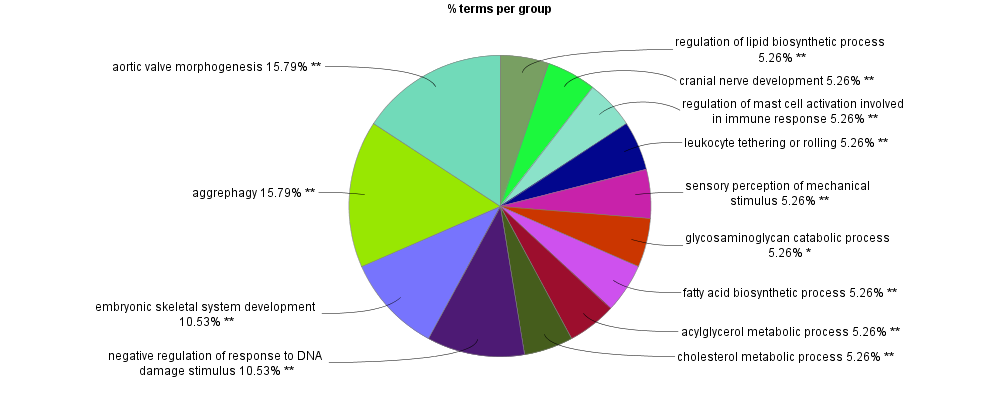

Supplement: Supplementary file 5 [file 41598_2020_58358_MOESM5_ESM.zip › Comparison 2/PieChart_terms.png]

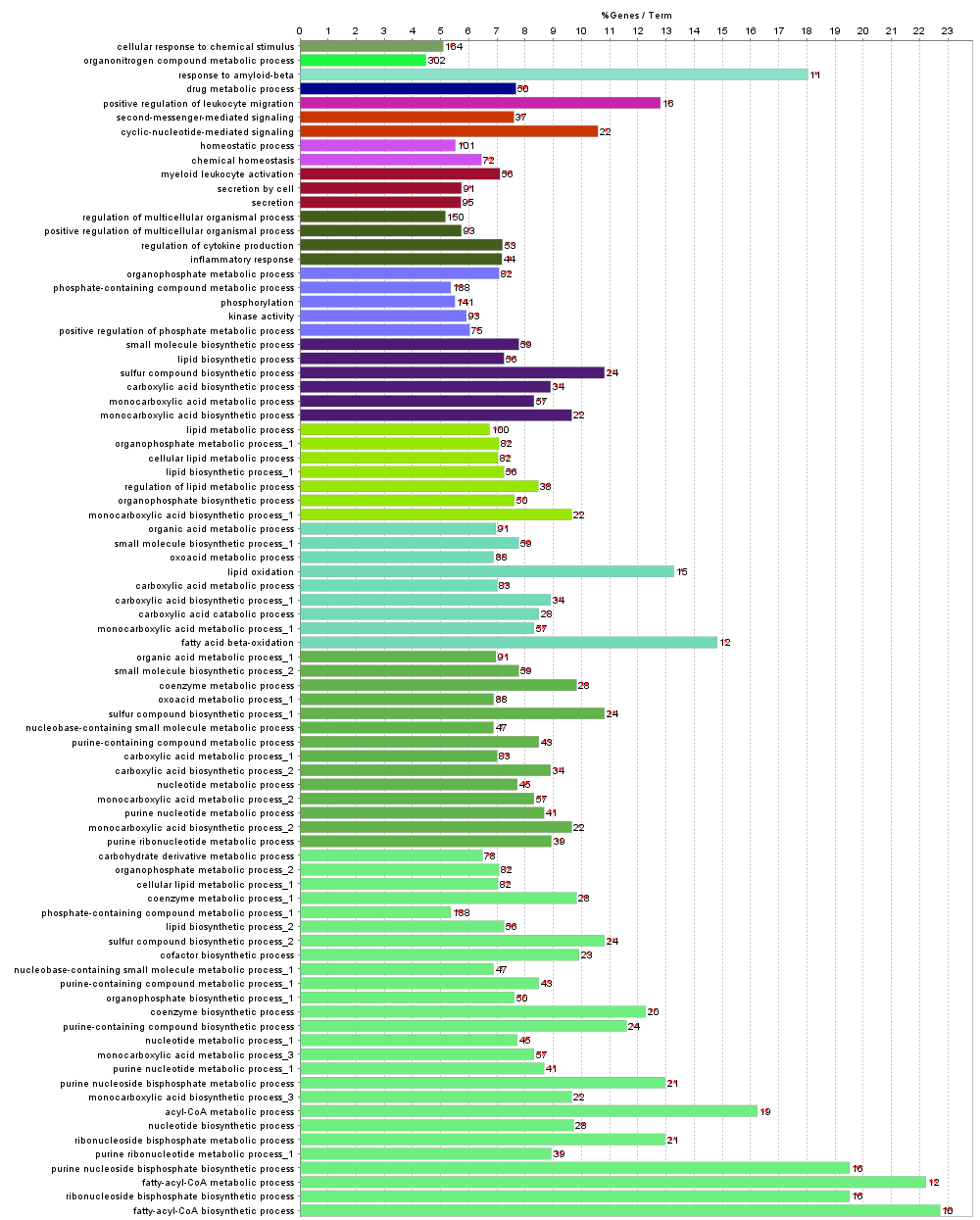

Supplement: Supplementary file 5 [file 41598_2020_58358_MOESM5_ESM.zip › Comparison 3/BarChart.png]

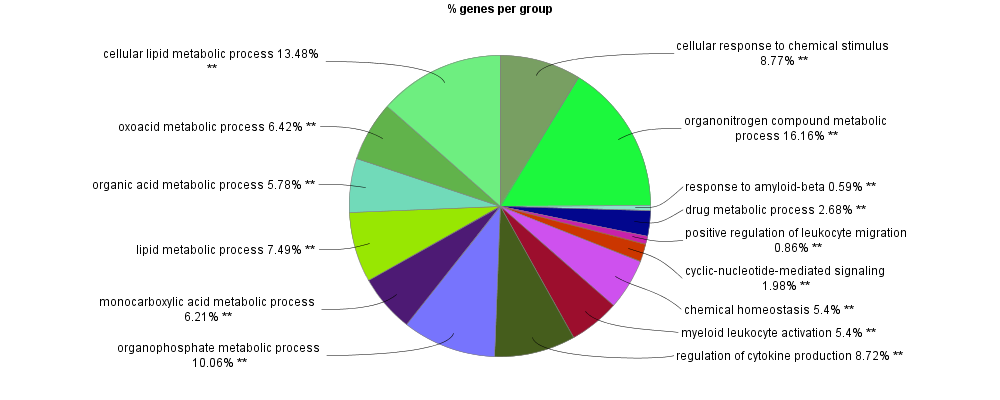

Supplement: Supplementary file 5 [file 41598_2020_58358_MOESM5_ESM.zip › Comparison 3/PieChart_genes.png]

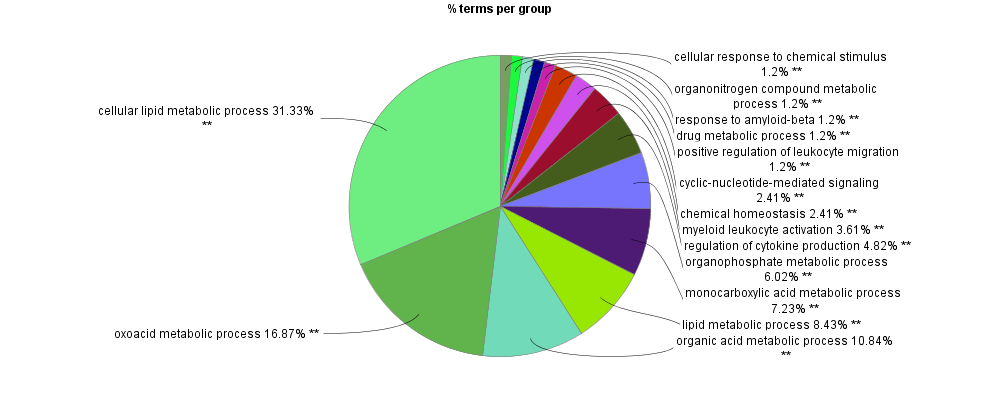

Supplement: Supplementary file 5 [file 41598_2020_58358_MOESM5_ESM.zip › Comparison 3/PieChart_terms.png]

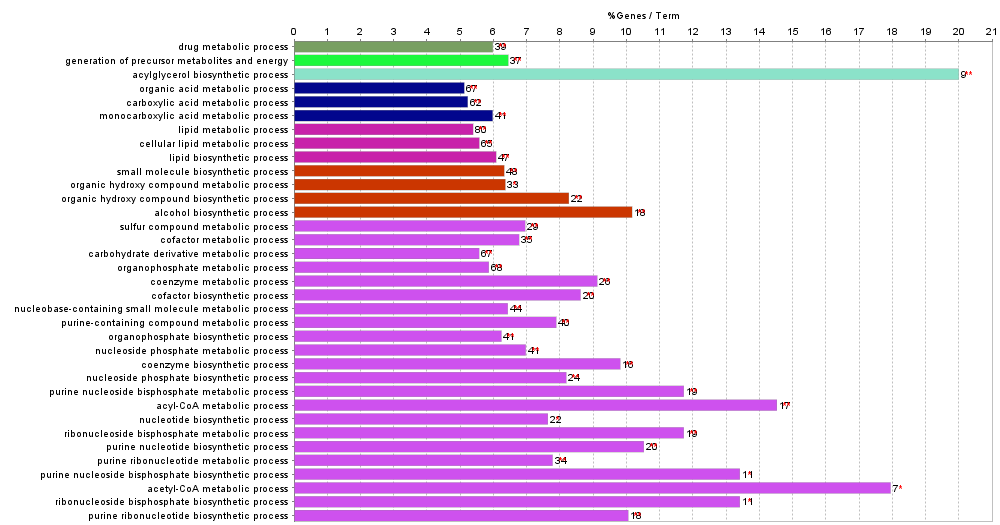

Supplement: Supplementary file 5 [file 41598_2020_58358_MOESM5_ESM.zip › Comparison 4/BarChart.png]

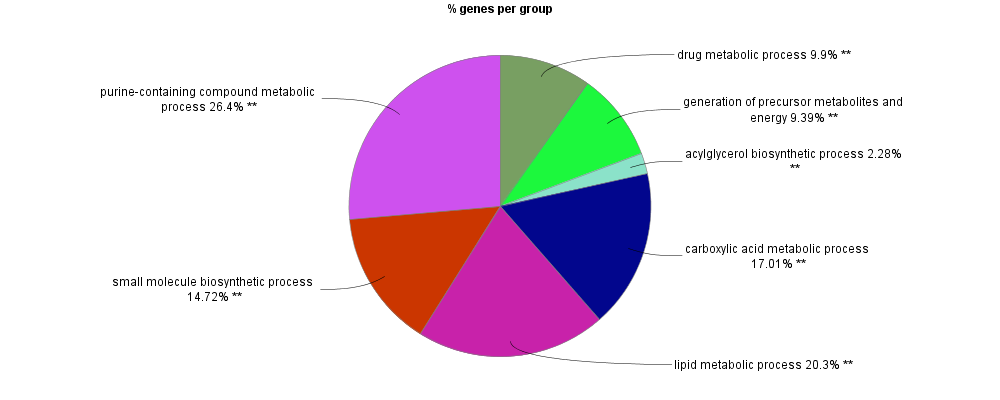

Supplement: Supplementary file 5 [file 41598_2020_58358_MOESM5_ESM.zip › Comparison 4/PieChart_genes.png]

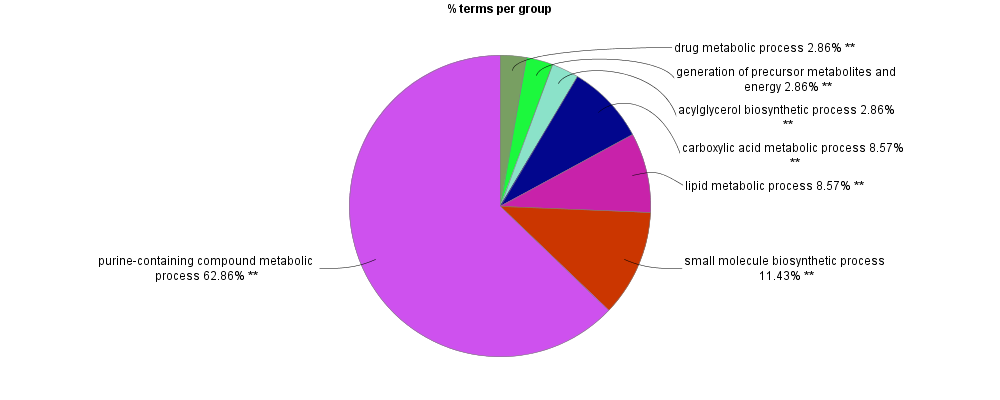

Supplement: Supplementary file 5 [file 41598_2020_58358_MOESM5_ESM.zip › Comparison 4/PieChart_terms.png]

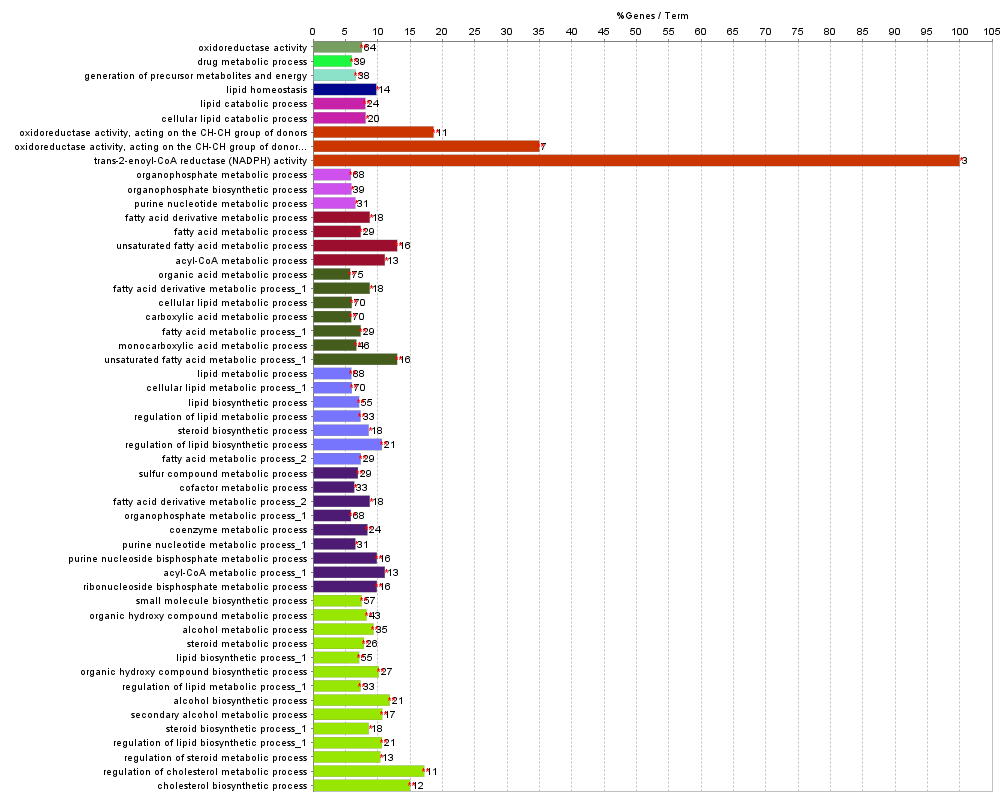

Supplement: Supplementary file 5 [file 41598_2020_58358_MOESM5_ESM.zip › Comparison 5/BarChart.png]

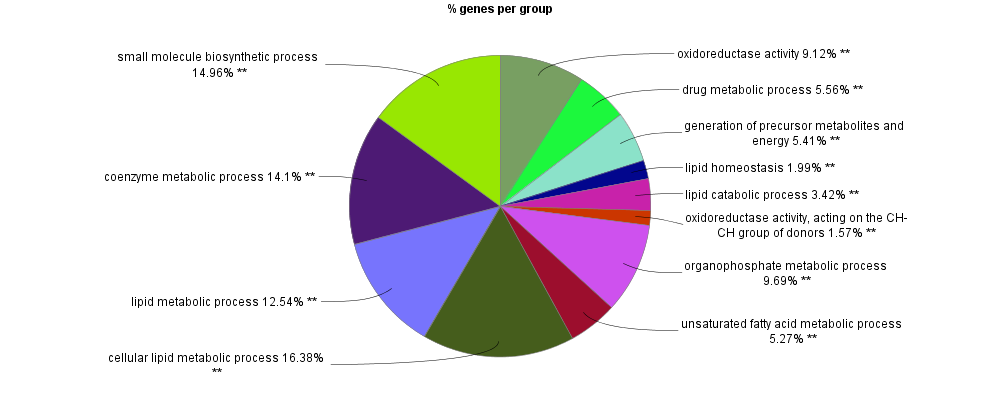

Supplement: Supplementary file 5 [file 41598_2020_58358_MOESM5_ESM.zip › Comparison 5/PieChart_genes.png]

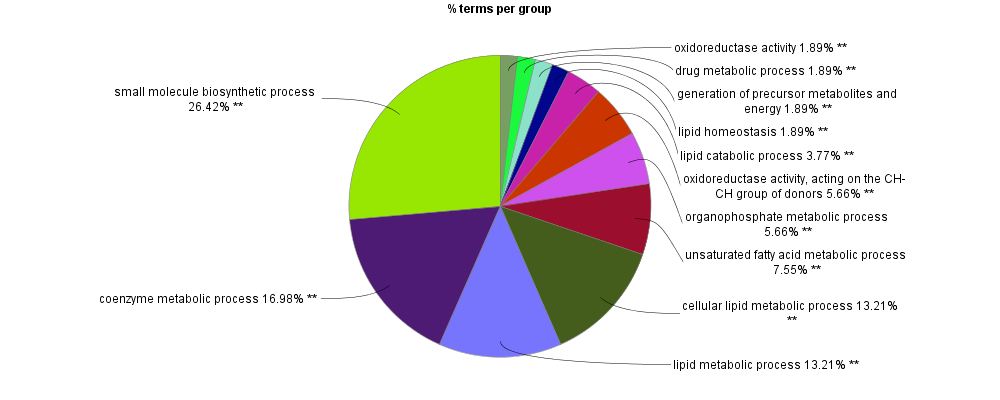

Supplement: Supplementary file 5 [file 41598_2020_58358_MOESM5_ESM.zip › Comparison 5/PieChart_terms.png]
